# Store-operated calcium entry via ORAI1 regulates doxorubicin-induced apoptosis and prevents cardiotoxicity in cardiac fibroblasts

**DOI:** 10.1371/journal.pone.0278613

**Published:** 2022-12-06

**Authors:** Hiroko Nemoto, Masanari Umemura, Fumina Suzuki, Akane Nagasako, Kagemichi Nagao, Yuko Hidaka, Rina Nakakaji, Keiji Uchida, Shinichi Suzuki, Munetaka Masuda, Yoshihiro Ishikawa

**Affiliations:** 1 Cardiovascular Research Institute, Yokohama City University Graduate School of Medicine, Yokohama, Kanagawa, Japan; 2 Department of Surgery, Yokohama City University Graduate School of Medicine, Yokohama, Kanagawa, Japan; Virginia Commonwealth University Medical Center, UNITED STATES

## Abstract

Despite exhibiting cardiotoxicity, doxorubicin (DOX) is widely used for cancer treatments. Cardiac fibroblasts (CFs) are important in the pathogenesis of heart failure. This necessitates the study of the effect of DOX on CFs. The impairment of calcium (Ca^2+^) homeostasis is a common mechanism of heart failure. Store-operated Ca^2+^ entry (SOCE) is a receptor-regulated Ca^2^⁺ entry pathway that maintains calcium balance by sensing reduced calcium stores in the endoplasmic reticulum. ORAI1, a calcium channel protein and the most important component of SOCE, is highly expressed in human cardiac fibroblasts (HCFs). It is upregulated in CFs from failing ventricles. However, whether ORAI1 in HCFs is increased and/or plays a role in DOX-induced cardiotoxicity remains unknown. In this study, we aimed to elucidate the relationship between ORAI1/SOCE and DOX-induced heart failure. Induction of apoptosis by DOX was characterized in HCFs. Apoptosis and cell cycle analyses were performed by fluorescence-activated cell sorting (FACS). Reactive oxygen species (ROS) production was measured using fluorescence. YM-58483 was used as an ORAI1/SOCE inhibitor. ORAI1-knockdown cells were established by RNA interference. In vivo experiments were performed by intraperitoneally injecting YM-58483 and DOX into mice. We first demonstrated that DOX significantly increased the protein expression level of p53 in HCFs by western blotting. FACS analysis revealed that DOX increased early apoptosis and induced cell cycle arrest in the G2 phase in fibroblasts. DOX also increased ROS production. DOX significantly increased the expression level of ORAI1 in CFs. Both YM-58483 and ORAI1 gene knockdown attenuated DOX-induced apoptosis. Similarly, YM-58483 attenuated cell cycle arrest in the G2 phase, and ORAI1 knockdown attenuated DOX-induced ROS production in HCFs. In the animal experiment, YM-58483 attenuated DOX-induced apoptosis. In HCFs, ORAI1/SOCE regulates p53 expression and plays an important role in DOX-induced cardiotoxicity. ORAI1 may serve as a new target for preventing DOX-induced heart failure.

## Introduction

Heart failure results from chemotherapy with anthracycline antibiotics. Doxorubicin (DOX) is a representative anthracycline antibiotic that is widely used to treat malignant lymphoma or breast cancer. However, heart failure develops in proportion to the cumulative dose of DOX [1]. In patients with breast cancer, DOX is often combined with trastuzumab, which may enhance cardiac toxicity [2]. DOX-induced heart failure is occasionally resistant to conventional pharmacological therapy with β-blockers, angiotensin-converting-enzyme inhibitors, or diuretics; thereby necessiating invasive therapies, such as ventricular assist devices or heart transplantation, for patients.

DOX induces oxidative stress in the cell membrane, which has been demonstrated using cardiomyocytes [3, 4]. Studies have identified other mechanisms of cardiotoxicity including DNA intercalation, topoisomerase II inhibition, apoptosis, mitochondrial dysfunction, autophagy, ferroptosis and inflammatory cytokines, and calcium homeostasis [5–9]. However, most of these investigations were performed using cardiac myocytes, and thus the involvement of other cell types was poorly characterized. Therefore, it is necessary to investigate the role of cardiac fibroblasts (CFs), which account for 60–70% of cells in the heart.

We previously reported that low doses of DOX did not induce myocyte death in mice but induced fibrosis localized to the perivascular area. Low-dose DOX induced reactive fibrosis and mitophagy through sterile inflammation evoked by the damaged mitochondria [10]. DOX also induced trans-differentiation of CFs to myofibroblasts and resulted in increased expression levels of matrix metalloproteinase 1 (MMP1), interleukin-6 (IL-6), transforming growth factor-β (TGF-β), and collagen, resulting in cardiac remodeling [11]. We also demonstrated that CFs reacted dynamically to physical stimuli such as hydrostatic pressure and/or hyperthermia [12, 13]. Therefore, CFs respond dynamically to various stresses, including DOX.

Store-operated-calcium entry (SOCE) is a receptor-regulated Ca^2+^ entry pathway that is activated by the depletion of intracellular Ca^2+^ stores. Specifically, stromal interaction molecule 1 (STIM1) senses the depletion of Ca^2+^ in the endoplasmic reticulum (ER). STIM1 then aggregates and moves to the plasma membrane to interact with ORAI1, a major Ca^2^⁺ channel protein involved in SOCE, and causes an influx of Ca^2^⁺ through the plasma membrane [14]. A recent study demonstrated that ORAI1, and thus SOCE, was upregulated in cardiac fibroblasts from failing ventricles [15], indicating the pivotal role played by CF ORAI1 in heart failure. However, whether ORAI1 is related to the progression of DOX-induced heart failure remains unknown.

In this study, we examined the relationship between DOX-induced heart failure and ORAI1/SOCE in CFs, which, to our knowledge, is unprecedented. We demonstrate that ORAI1/SOCE may be a potential new target for the prevention and treatment of DOX-induced heart failure.

## Materials and methods

### Reagents

DOX hydrochloride was purchased from Sigma-Aldrich (MO, USA). In the in vitro experiments, DOX was added directly to the medium. N-[4-[3,5-bis(trifluoromethyl)-1H-pyrazol-1-yl]phenyl]-4-methyl-1,2,3-thiadiazole-5-carboxamide (YM-58483) (also known as BTP2) was used as a SOCE inhibitor and was purchased from Cayman Chemical (MI, USA). Cells were incubated with or without YM-58483 1 h prior to DOX administration. Other reagents are described in the applicable sections.

### Cell lines

Human cardiac fibroblasts (HCFs) were purchased from ScienCell Research Laboratories (CA, USA). HCFs were cultured in fibroblast medium-2 (FM-2) (ScienCell Research Laboratories, CA, USA), a commercial fibroblast medium supplemented with 1% penicillin/streptomycin, 1% fibroblast growth supplement-2, and 2% foetal bovine serum. All cells were maintained in a humidified atmosphere of 95% air and 5% CO_2_ at 37°C. Passages 4–9 were used for the experiments. HCFs were serum starved for 16 h before the administration of YM-58483 or DOX.

### ORAI1 knockdown via RNA interference

Small interfering (si) RNA against ORAI1 (ON-TARGET plus ORAI1 siRNA SMART pool, L-014998-00-0005) and control siRNA (ON-TARGET plus non-targeting siRNA#1, D-001819-01-05) were purchased from Dharmacon (CO, USA). Target sequences of ORAI1 siRNA were as follows: GGCCUGAUCUUUAUCGUCU (ON-TARGET plus SMARTpool siRNA J-014998-05), GCACCUGUUUGCGCUCAUG (ON-TARGET plus SMARTpool siRNA J-014998-06), GGAGUUUGCCCGCUUACAG (ON-TARGET plus SMARTpool siRNA J-014998-07), and UCAACGAGCACUCCAUGCA (ON-TARGET plus SMARTpool siRNA J-014998-08).

These siRNAs were transfected into HCFs using OPTI-MEM (Thermo Fisher Scientific, MA, USA) containing Lipofectamine RNAiMAX Reagent (Invitrogen, CA, USA). HCFs were assayed for efficacy of ORAI1 knockdown following incubation for 72 h.

### Apoptosis assay

HCFs were incubated with or without 1.0 μM DOX for 24 h. A FITC Annexin V Apoptosis Detection Kit with PI (BioLegend, CA, USA) was used to analyze apoptosis. HCFs were labeled with annexin V–FITC and propidium iodide. The cells that underwent early and late apoptosis were counted by flow cytometry using BD fluorescence-activated cell sorting (FACS) Canto II (BD Biosciences, CA, USA). Cells were divided into four sections: Q1–Q4. Normal cells were located in Q4, early apoptotic cells in Q3, and late apoptotic or necrotic cells in Q2. Considering the fluorescence of DOX, FACS was used to measure cells without annexin V–FITC and propidium iodide as a negative control for each sample. We analyzed early apoptotic cells by subtracting the negative control from Q3.

### Cell cycle assay

HCFs were incubated with or without 1.0 μM DOX for 24 h. BD Cycletest Plus DNA Reagent Kit (BD Bioscience) was used for cell cycle analysis. Cell cycle distributions were measured using BD FACS Canto II (BD Biosciences).

### Mitochondrial reactive oxygen species measured by fluorescence

Reactive oxygen species (ROS) production was measured by fluorescence. For fluorescence measurement, HCFs were incubated with or without DOX (0.2 to 5.0 μM) for 24 h. Cells were superfused with 2-(2,7-dichloro-3,6-diacetyloxy-9H-xanthen-9-yl)-benzoic acid (DCFH-DA) (Cayman Chemical, MI, USA) in a serum-free medium for 40 min at 37°C. Staining intensity was measured using a microplate reader (PerkinElmer, MA, USA). Fluorescence signals obtained at 485 and 535 nm were measured with a fluorescence system.

### Quantitative real-time reverse transcription polymerase chain reaction

Total RNA from HCFs was extracted using the ISOSPIN Cell & Tissue RNA kit (Nippon Gene, Tokyo, Japan). Reverse transcription reactions were performed using the PrimeScript RT reagent kit (TaKaRa Bio, Shiga, Japan). Quantitative polymerase chain reactions (qPCR) were prepared using Taqman qPCR Mix (TaKaRa Bio). Reverse transcription–polymerase chain reactions were performed on the StepOnePlus Real-Time PCR System (Applied Biosystems, Waltham, MA, USA). The 2 ΔΔCt method was used to determine relative gene expression levels and the 18S rRNA gene was used to normalize the data. The sequences of the specific primers were deposited in the Applied Biosystems database under description ID Hs00385627_m1 (ORAI1) and Hs99999905_m1 (18S).

### Western blotting

Western blot analyses were performed in accordance to a method in a previous study [16] and in S1 Table. Briefly, HCFs were stimulated using DOX (0.1 to 1.0 μM) for 1–24 h. Cells were lysed and sonicated in RIPA buffer (Thermo Fisher Scientific, IL, USA). Equal amounts of protein were subjected to sodium dodecyl sulfate polyacrylamide gel electrophoresis (SDS-PAGE). For electrophoretic separation, AllView PAGE Buffer (BioDynamics Laboratory Inc., Tokyo, Japan) or 1× loading buffer were used. After electrophoretic separation, protein bands were transferred to a Millipore Immobilon-P membrane followed by immunoblotting with the following primary antibodies: anti-STIM1 (Cell Signaling Technology, MA, USA; 1:1000), anti-ORAI1 (Sigma Aldrich, MO, USA; 1:1000), anti-p53 (Cell Signaling Technology; 1:1000), anti-p21 Waf1/Cip1 (12D1) (Cell Signaling Technology; 1:1000), and anti-glyceraldehyde 3-phosphate dehydrogenase (GAPDH) (Cell Signaling Technology; 1:4000). The GAPDH antibody was used as a loading control to normalize the data. The following secondary antibody was then used for immunoblotting: anti-rabbit IgG, HRP-linked antibody (Cell Signaling Technology; 1:4000 for use against STIM1, p53, p21 and GAPDH antibodies, and 1:2000 for use against ORAI1 antibody). Chemiluminescence detection was performed using the ECL reagent (Bio-Rad Laboratories, CA, USA). Signals were revealed using a LuminoGraph II (ATTO, Tokyo, Japan). Signal intensities of the bands were quantified using ATTO CS Analyzer 4 software (ATTO).

### Animal study

Eight-week-old male C57BL/6J mice were purchased from Japan SLC (Shizuoka, Japan) .28 mice were fed at the Laboratory Animal Center of Yokohama City University School of Medicine for 1 week. All staffs were provided special training in animal care or handling. Mice were randomly allocated into four groups (n = 7 per group) as follows: control group (CTRL group), DOX-treated group (DOX group), YM-58483-treated group (YM group), and YM-58483 and DOX-treated group (YM+DOX group). DOX was intraperitoneally administered at 40 mg/kg once. YM-58483 was administered intraperitoneally 1 h prior to DOX administration. After administering DOX, seven mice from each group were divided into two cages (three mice and four mice) to be fed. Animal health and behabior were monitored every 12 h. At 48 h after DOX administration, all mice were alive. They were then euthanized by drawing blood from inferior vena cava under inhalation anesthesia with 5% isoflurane. In a state of resuscitation difficulty, the hearts were rapidly excised and the left ventricles were fixed in 10% formalin.

### Histological analysis

Left ventricle tissues were fixed in 10% formalin, embedded in paraffin, and sectioned at a thickness of 3.5 μm. Sections were then deparaffinized. Apoptosis in the cardiac tissue was evaluated using the terminal deoxynucleotidyl transferase-mediated dUTP nick-end labelling (TUNEL) assay with the DeadEnd Fluorometric TUNEL System (Promega, Madison, WI, USA) as described in a previous study [10]. Nuclei were stained with 4’,6-diamidino-2-phenylindole (DAPI). Cardiac tissues were then visualized using fluorescence microscopy with an inverted BZ-X810 microscope (Keyence, Osaka, Japan). Five random fields were selected for analysis and TUNEL-positive nuclei and total nuclei were counted.

### Ethics statement

Animal studies were carried out in strict accordance with the recommendations in the Guide for the Care and Use of Laboratory Animals of the National Institutes of Health. All animal studies and experimental protocols were approved by the Animal Care and Use Committee at Yokohama City University School of Medicine (Protocol Number: F-A-20-079). All surgeries were performed under inhalation anesthesia with 5% isoflurane, and every effort was made to minimize suffering. From humane endpoints, if mice become debilitating based on anticipated clinical, physiological, and behavioral signs including weight changes, abnormal behaviors, reduced mobility, body posture in the middle of observation for 48 h, we had the criteria to euthanized them promptly (within 30 min) with carbon dioxide gas. No animals died before meeting this criteria for euthanasia.

### Data analysis and statistics

Statistical analysis was performed using GraphPad Prism 9 software (GraphPad Software Inc., San Diego, CA, USA). Statistical comparisons were performed between two groups using the Student’s t-test. Multiple comparisons were made using one-way analysis of variance (ANOVA) followed by Tukey’s test or two-way ANOVA followed by Bonferroni’s post hoc test. Statistical significance was set at *p* < 0.05.

## Results

### Doxorubicin increased expression level of p53

**Fig 1:**
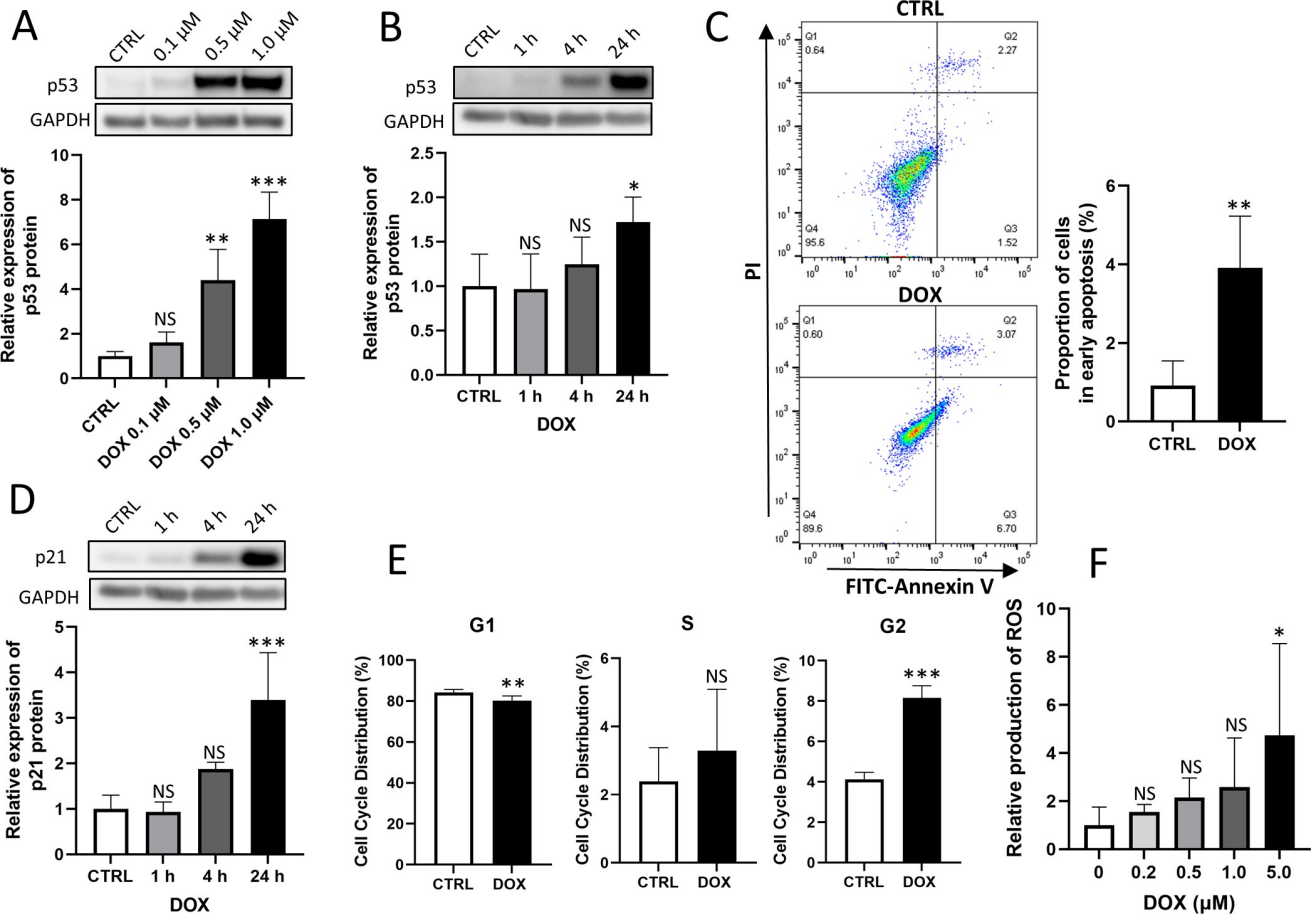
**1A and 1B. Western blotting analysis comparing doxorubicin (DOX) samples with control (CTRL) samples.** (A) Human cardiac fibroblasts (HCFs) were exposed to 0.1 to 1.0 μM DOX for 24 h. DOX increased the expression of p53 protein in a dose-dependent manner (one-way ANOVA followed by Tukey’s test, n = 4, *** *p* < 0.001, ** *p* < 0.01, NS: no significant difference). (B) HCFs were exposed to 0.5 μM DOX for 1 to 24 h. DOX increased the expression of p53 protein in a time-dependent manner (one-way ANOVA followed by Tukey’s test, n = 4, * *p* < 0.05, NS: no significant difference). **1C. Apoptosis assay comparing DOX samples with CTRL samples.** Flow cytometry analysis showed that DOX significantly increased early apoptosis (Q3) (unpaired t-test, n = 4, ** *p* < 0.01). **1D and 1E. Cell cycle analysis comparing DOX samples with CTRL samples.** (D) Western blotting analysis. HCFs were exposed to 0.5 μM DOX for 1 to 24 h. DOX increased the expression of p21 protein in a time-dependent manner (one-way ANOVA followed by Tukey’s test, n = 4, *** *p* < 0.001, NS: no significant difference). (E) Cell cycle assay by FACS showed that DOX significantly decreased the proportion of cells in the G1 phase and increased those in the G2 phase of the cell cycle (unpaired t-test, n = 6, *** *p* < 0.001, ** *p* < 0.01, NS: no significant difference). **1F. ROS production measured by fluorescence 24 h after administration of DOX.** DOX increased ROS production significantly in the samples with 5.0 μM DOX (one-way ANOVA followed by Tukey’s test, n = 6, * *p* < 0.05, NS: no significant difference).

p53 responds to various cardiac stresses [17–19] and plays a major role in cardiac apoptosis [20, 21], cell proliferation [22], and ROS production [23]. Upregulation of p53 and thus cardiac fibrosis have been observed in heart failure associated with dilated cardiomyopathy [24]. p53 levels were observed to increase in DOX-treated cardiac myocytes [25]. We therefore examined whether DOX increased p53 levels in cultured HCFs rather than cardiac myocytes. As shown in Fig 1A and 1B, DOX significantly increased the expression level of p53 protein in HCFs in both dose- and -time-dependent manners.

### Doxorubicin increased apoptosis in human cardiac fibroblasts

As p53 activation occurs with apoptosis induced by DOX in cardiac myocytes [26, 27], we examined the development of apoptosis in HCFs using flow cytometry by FACS. HCFs were treated with 1.0 μM DOX at 37°C for 24 h, followed by labeling with annexin V–FITC and propidium iodide. We found that early apoptosis increased significantly upon treatment with DOX (Fig 1C).

### Doxorubicin promoted cell cycle arrest in human cardiac fibroblasts

The upregulated p53 expression level increases the transcription of p21, leading to cell cycle arrest in the G2/M phase [22]. We found that DOX significantly increased the protein expression levels of p21 (Fig 1D). FACS showed that DOX significantly decreased the proportion of cells in the G1 phase of the cell cycle and increased those in the G2 phase (Fig 1E), suggesting that DOX increased the levels of p21 and arrested HCFs in the G2 phase.

### Doxorubicin increased production of reactive oxygen species in human cardiac fibroblasts

DOX is known to increase ROS production in cardiac myocytes [28]. DOX also increased ROS production in HCFs in a dose-dependent manner, as measured using fluorescence (Fig 1F).

### Inhibition of ORAI1/SOCE reduced doxorubicin-induced expression of p53 and p21

**Fig 2:**
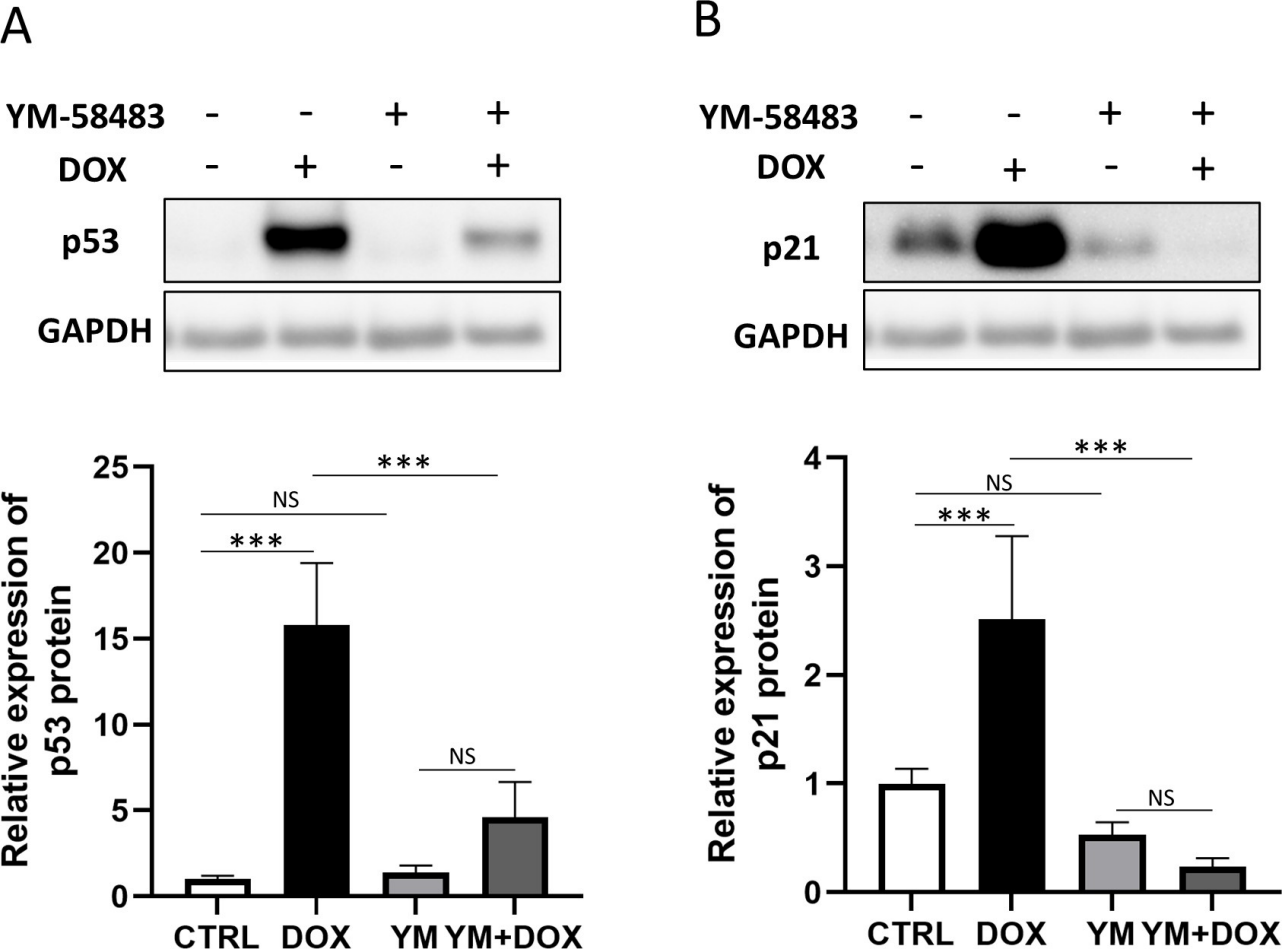
Western blotting analysis for four groups to evaluate the effects of store-operated Ca^2+^ entry (SOCE) inhibition; CTRL group, DOX group, YM group, and YM+DOX group. (A) YM-58483 significantly attenuated the DOX-induced upregulation of p53 protein. (B) YM-58483 significantly attenuated the DOX-induced upregulation of p21 protein. (one-way ANOVA followed by Tukey’s test, n = 6, *** *p* < 0.001, NS: no significant difference).

ORAI1, a major component of SOCE, is a Ca^2+^ channel protein that is highly expressed in ventricular fibroblasts from failing human hearts [15]. We therefore examined whether ORAI1 is involved in the DOX-induced changes in HCFs. YM-58483, an inhibitor of SOCE, attenuated the DOX-induced upregulation of p53 (Fig 2A). Similarly, the upregulation of p21 was attenuated in the presence of YM-58483 (Fig 2B). Therefore, the upregulation of both p53 and p21 were attenuated by the pharmacological inhibition of ORAI1.

**Fig 3:**
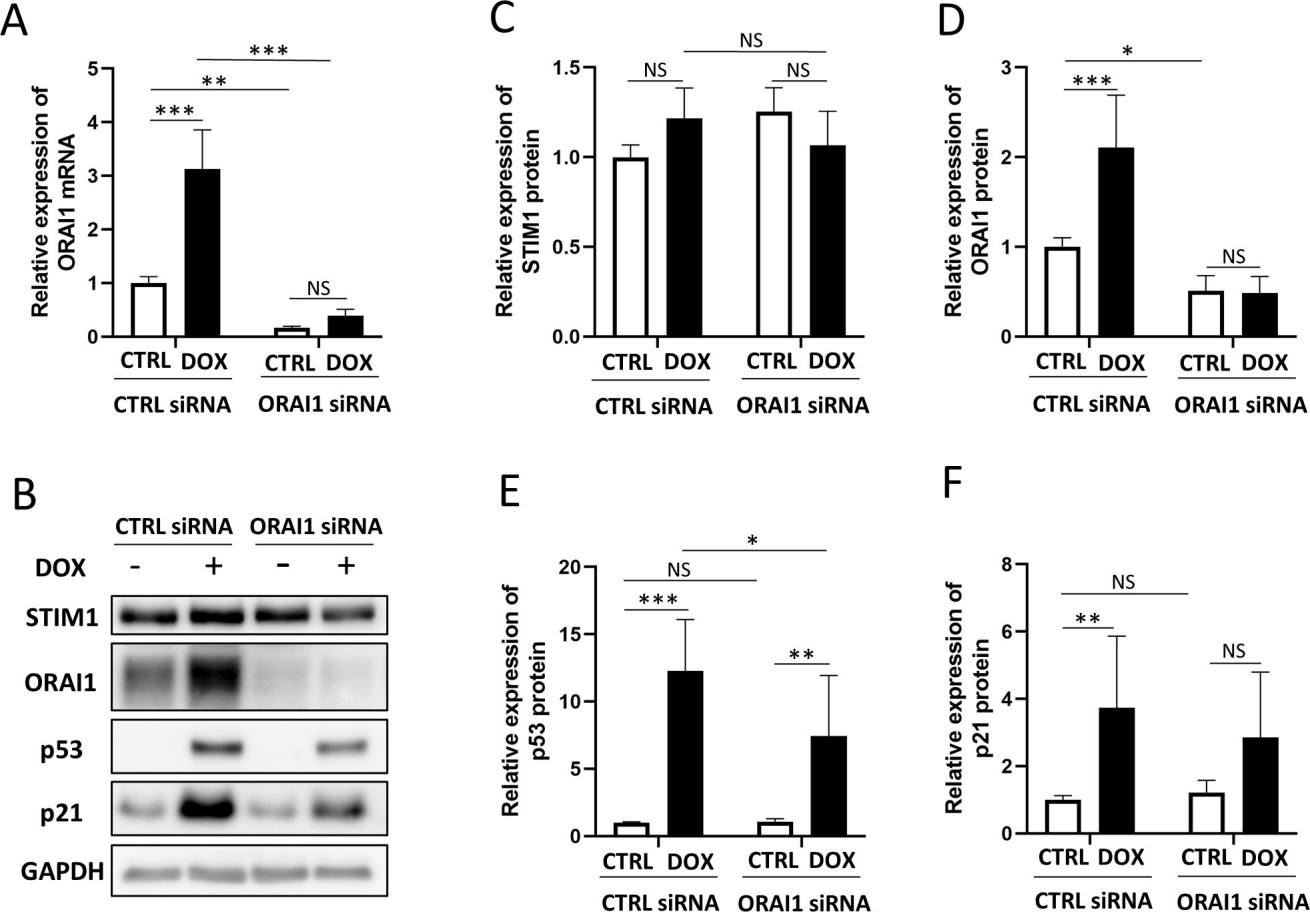
Western blotting analysis to evaluate the effects of ORAI1 gene knockdown. (A) Expression of ORAI1 mRNA by qPCR. The expression level of ORAI1 mRNA was reduced in samples with siRNA against ORAI1. In samples with control siRNA, DOX significantly upregulated the expression level of ORAI1 mRNA (two-way ANOVA followed by Bonferroni’s post hoc test, n = 6, *** *p* < 0.001, ** *p* < 0.01, NS: no significant difference). (B) Western blotting of four groups; CTRL siRNA group without DOX, CTRL siRNA group with DOX, ORAI1 gene knockdown (KD) group without DOX, and ORAI1 gene KD group with DOX. (C) There were no significant differences in the expression of STIM1 protein (two-way ANOVA followed by Bonferroni’s post hoc test, n = 7, NS: no significant difference). (D) Knockdown of the ORAI1 gene eliminated the expression of ORAI1 protein. DOX upregulated the expression level of ORAI1, but there was no significant difference in the samples with ORAI1 KD siRNA (two-way ANOVA followed by Bonferroni’s post hoc test, n = 7, *** *p* < 0.001, * *p* < 0.05, NS: no significant difference). (E) Knockdown of ORAI1 gene negated DOX-induced expression of p53 protein (two-way ANOVA followed by Bonferroni’s post hoc test, n = 8, *** *p* < 0.001, ** *p* < 0.01, * *p* < 0.05, NS: no significant difference). (F) In the samples with control siRNA, DOX upregulated the expression level of p21 protein. No significant difference was identified in samples with ORAI1 KD siRNA (two-way ANOVA followed by Bonferroni’s post hoc test, n = 8, ** *p* < 0.01, NS: no significant difference).

Next, we established an ORAI1 gene knockdown model by transfecting siRNA into HCFs. ORAI1 gene knockdown was confirmed by measuring ORAI1 levels by qPCR (Fig 3A) and western blotting (Fig 3B). While there was no significant difference in the expression level of STIM1 protein (Fig 3C), DOX significantly increased the expression level of ORAI1 protein in CFs. This increase was attenuated by ORAI1 gene knockdown (Fig 3D). The ORAI1 gene knockdown also attenuated the DOX-induced upregulation of p53 (Fig 3E). Similarly, DOX increased the expression level of p21 protein, and this increase was attenuated by ORAI1 gene knockdown (Fig 3F).

These results suggest that ORAI1/SOCE may play an important role in promoting apoptosis, cell cycle arrest, and ROS production induced by DOX.

### Inhibition of ORAI1/store-operated Ca^2+^ entry attenuated doxorubicin-induced apoptosis

**Fig 4:**
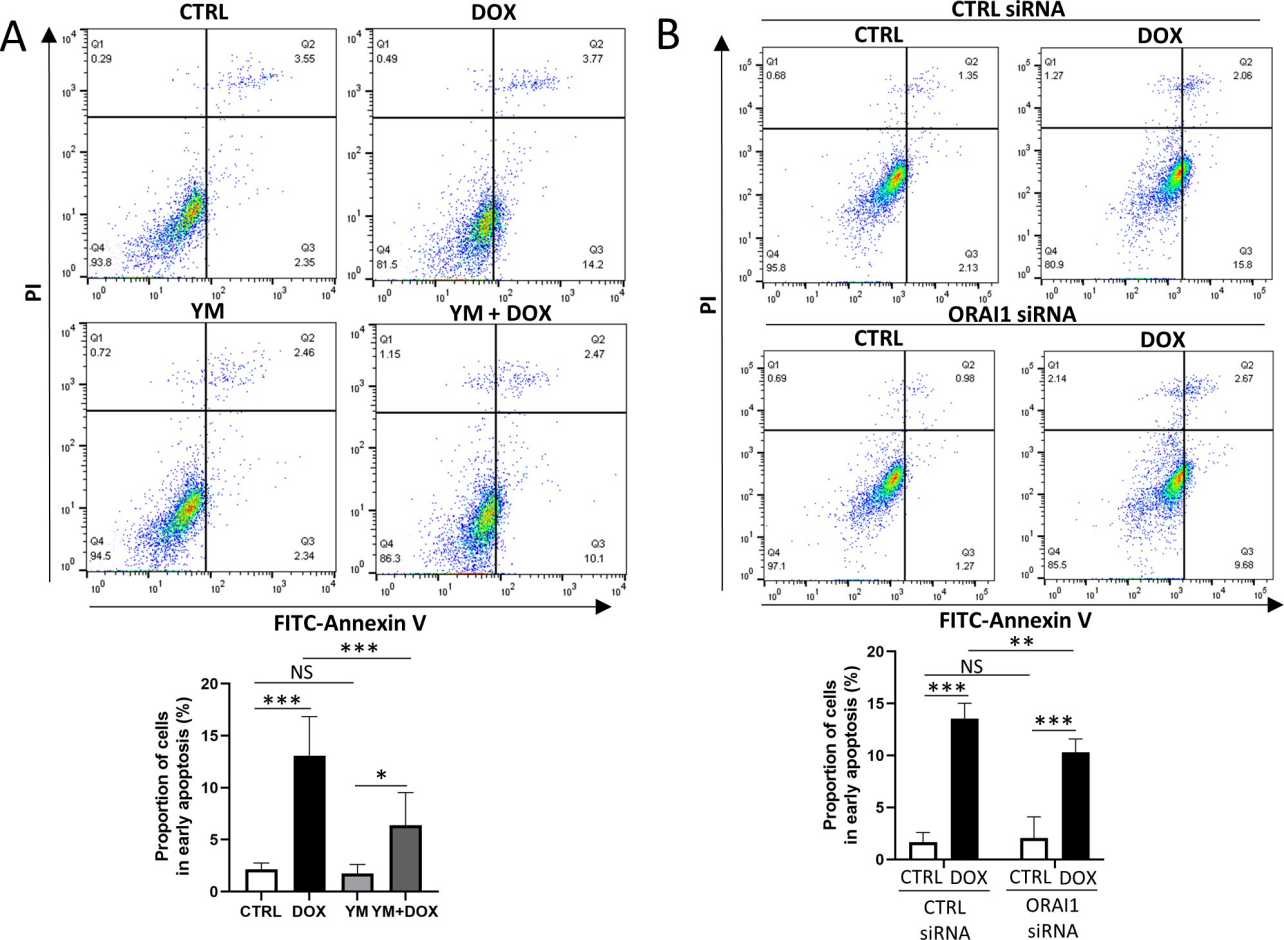
Apoptosis assay to evaluate the effects of SOCE inhibition. (A) Apoptosis assay of four groups: CTRL group, DOX group, YM group, and YM+DOX group. Flow cytometry showed that YM-58483 significantly attenuated DOX-induced early apoptosis (one-way ANOVA followed by Tukey’s test, n = 6, *** *p* < 0.001, * *p* < 0.05, NS: no significant difference). (B) Apoptosis assay of four groups: CTRL siRNA group without DOX, CTRL siRNA group with DOX, ORAI1 KD siRNA group without DOX, and ORAI1 KD siRNA group with DOX. Flow cytometry showed that the knockdown of the ORAI1 gene significantly attenuated DOX-induced early apoptosis (two-way ANOVA followed by Bonferroni’s post hoc test, n = 6, *** *p* < 0.001, ** *p* < 0.01, NS: no significant difference).

DOX induces ER stress and activates apoptosis in the heart [29], We therefore hypothesized that DOX stimulates SOCE, leading to enhanced ER stress and early apoptosis. To assess this, we performed flow cytometry using FACS in four groups: the CTRL group, DOX group, YM group, and YM+DOX group. The proportion of cells in early apoptosis significantly decreased in the YM+DOX group compared to that in the DOX group (Fig 4A).

We also evaluated the effect of ORAI1 gene knockdown on apoptosis. Early apoptosis was significantly increased in the CTRL siRNA groups while the magnitude of early apoptosis was significantly decreased in the ORAI1 knockdown siRNA groups (Fig 4B). Therefore, pharmacological inhibition of SOCE by YM-58483 and genetic inhibition by knockdown of the ORAI1 gene attenuated DOX-induced apoptosis in HCFs.

### Inhibition of ORAI1/store-operated Ca^2+^ entry attenuated doxorubicin-induced cell cycle arrest

To evaluate the effects of ORAI1/SOCE inhibition on DOX-induced cell cycle arrest, we performed flow cytometry using FACS in four groups: CTRL, DOX, YM, and YM+DOX. FACS showed that YM-58483 increased the proportion of cells in the G1 phase, which was suppressed by DOX. Conversely, YM-58483 tended to decrease the proportion of cells in the G2 phase, which was increased by DOX (S1 Fig). These results suggest that the inhibition of ORAI1/SOCE by YM-58483 decreased DOX-induced cell cycle arrest in HCFs.

### Inhibition of ORAI1/store-operated Ca^2+^ entry attenuated doxorubicin-induced reactive oxygen species production

**Fig 5:**
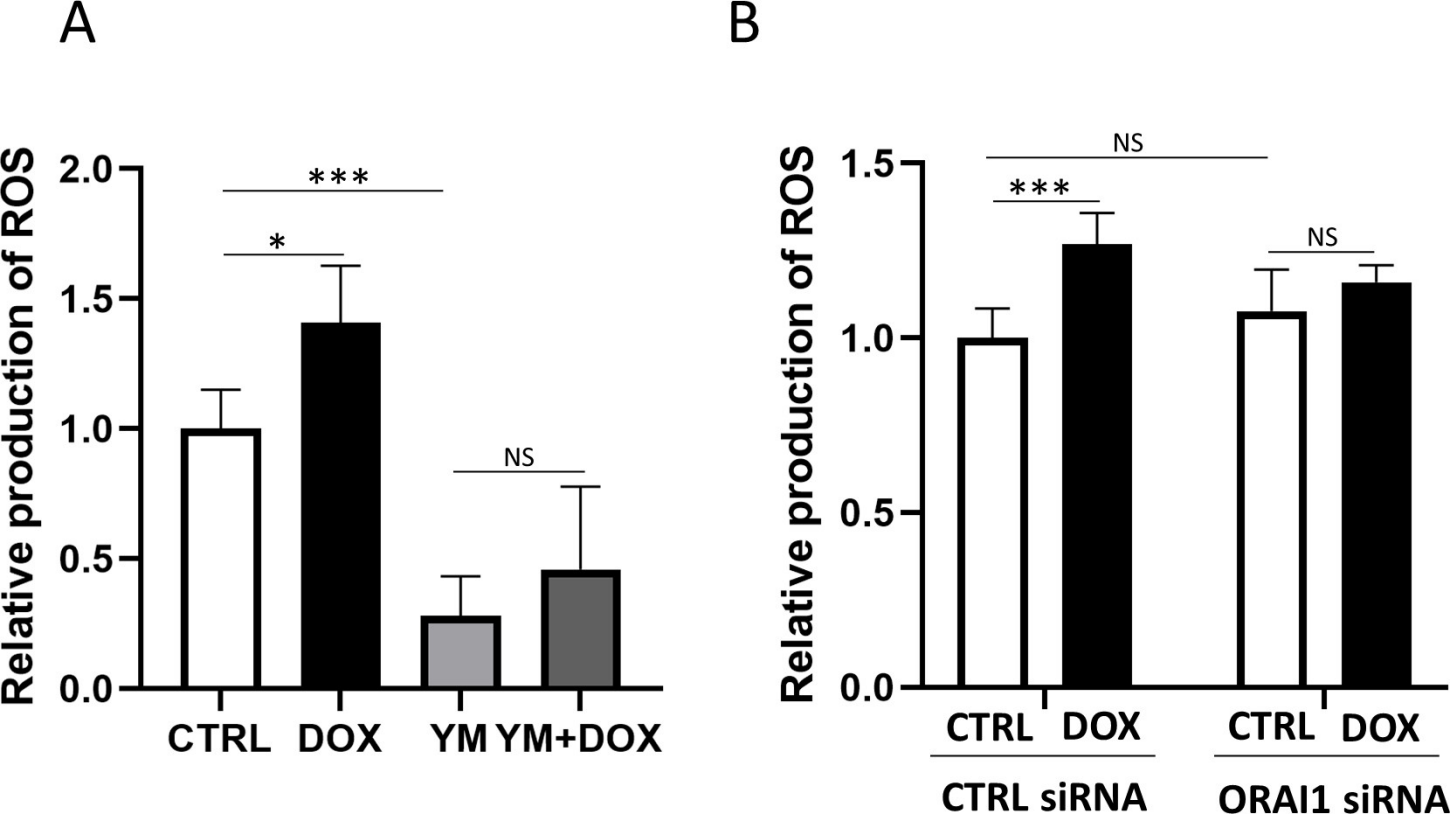
ROS production measured by fluorescence to evaluate the effects of SOCE inhibition. (A) Four groups were analyzed: the CTRL group, DOX group, YM group, and YM+DOX group. DOX significantly increased ROS production. There were no significant differences among the ROS productions of YM-58483-treated groups. (one-way ANOVA followed by Tukey’s test, n = 6, *** *p* < 0.001, * *p* < 0.05, NS: no significant difference). (B) Four groups were analyzed: CTRL siRNA group without DOX, CTRL siRNA group with DOX, ORAI1 KD siRNA group without DOX, and ORAI1 KD siRNA with DOX. In the CTRL siRNA groups, DOX significantly increased ROS production in both conditions. In the ORAI1 knockdown siRNA groups, there was no significant difference in ROS production between the with DOX and without DOX groups (two-way ANOVA followed by Bonferroni’s post hoc test, n = 6, *** *p* < 0.001, NS: no significant difference).

To evaluate the effects of ORAI1/SOCE inhibition on DOX-induced ROS production, we measured ROS using fluorescein in four groups: the CTRL group, DOX group, YM group, and YM+DOX group (Fig 5A). DOX significantly increased ROS production, but there was no significant difference between the YM and YM+DOX groups.

We then compared DOX-induced ROS production between CTRL siRNA groups and ORAI1 knockdown siRNA groups (Fig 5B). In the CTRL siRNA groups, DOX significantly increased ROS production while no significant difference was observed in the ORAI1 knockdown groups. These results suggest that the inhibition of ORAI1/SOCE attenuated DOX-induced ROS production.

### Doxorubicin increased cardiac apoptosis, which was attenuated by YM-58483 in mice

**Fig 6:**
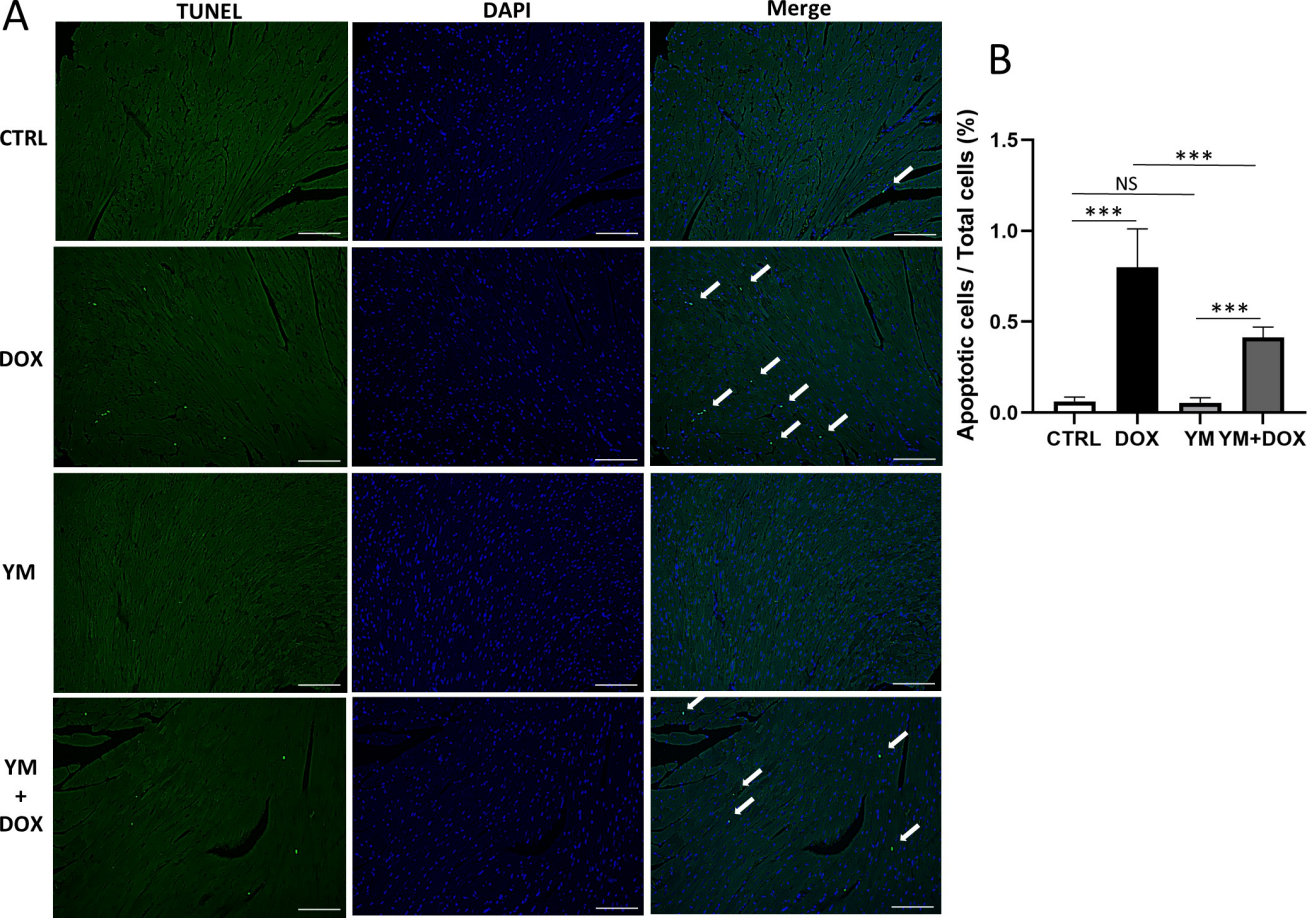
Assessment of DOX-induced cardiotoxicity in mice. Saline or 40 mg/kg DOX was administered intraperitoneally. In addition, 0.2% DMSO or 10 mg/kg YM-58483 was administered intraperitoneally prior to saline or DOX. (A) TUNEL apoptosis staining in heart sections. Apoptotic nuclei were quantitatively assessed in five random fields. Scale bar, 100 μm. (B) YM-58483 significantly attenuated DOX-induced apoptosis (one-way ANOVA, n = 7, *** *p* < 0.001, NS: no significant difference).

We previously reported that low doses of DOX did not induce apoptosis, and that fibrosis was localized to the perivascular area in mice [10]. In this study, a single toxic dose (40 mg/kg) of DOX was administered, followed by TUNEL staining. We administered YM-58483 intraperitoneally at 10 mg/kg 1 h prior to DOX administration. We compared apoptotic changes in four groups: the CTRL group, DOX group, YM group, and YM+DOX group. In the DOX group, the number of apoptotic cells significantly increased while YM-58483 significantly attenuated DOX-induced apoptosis (Fig 6A and 6B). Therefore, DOX-induced cardiotoxicity was attenuated by ORAI1/SOCE inhibition.

## Discussion

In the current study, we demonstrated that DOX increased p53 and p21 levels in HCFs. FACS analysis revealed increments in early apoptosis and cell cycle arrest in the G2 phase. DOX increased ROS production in HCFs. Inhibition of ORAI1 attenuated the DOX-induced apoptosis, cell cycle arrest, ROS production, and changes in p53 and p21 levels in HCFs. Furthermore, YM-58483 attenuated DOX-induced apoptosis in vivo in mice. Our study strongly suggests that ORAI1 plays an important role in DOX-induced cardiotoxicity in HCFs and that ORAI1 may serve as a new target for preventing DOX-induced cardiac toxicity. To our knowledge, this is the first study that demonstrates the role of p53 and ORAI1/SOCE in DOX-induced toxicity in CFs (S2 Fig).

DOX induces heart failure in a dose-dependent manner. Many studies have addressed ways of preventing such DOX toxicity as it leads to inferior prognosis [5]. For example, carvedilol exhibits a potentially protective effect by decreasing the release of free radicals into cardiomyocytes [30]. In addition, beta-hydroxybutyrate may exert protective effects by inhibiting oxidative stress and maintaining the integrity of the mitochondrial membrane [31]. Mangiferin, a naturally occurring glucosylxanthone, shows antioxidant and cardioprotective properties via the regulation of intracellular calcium movement [32]. We examined the role of ORAI1/SOCE as a new mechanism for the prevention of DOX-induced heart failure.

To address this, we examined the relationship between DOX and p53, which is a well-known tumor supressor involved in apoptosis, cell cycle arrest, and ROS production. The absence of p53 increases susceptibility to DOX-induced cardiotoxicity while a mutant of p53 that retains mitochondrial regulation was found to be protective [19]. The use of valsaltan, an angiotensin-II receptor blocker, showed a marked decrease in levels of cardiac biomarker enzymes (aminotransferase and creatine phosphokinase) and fibrosis, as well as p53 and Bax expression [33]. Although these studies have suggested the potential involvement of CFs in the pathogenesis of DOX-induced cardiac toxicity, few studies have investigated DOX-induced changes in p53 levels in CFs. CFs are essential in extracellular matrix production, cardiac remodeling, and modulating the myocardial response to changes in electrical and chemical signaling [34]. Mancilla et al. suggested that DOX increased the expression level of p53 and induced mitophagy by preventing the localization of Parkin in CFs [35]. Despite a previous study showing that p53 overexpression in normal pulmonary arterial smooth muscle cells inhibits SOCE, while downregulation of p53 enhanced SOCE [36], no studies have demonstrated that ORAI1/SOCE interferes with p53 expression.

YM-58483/BTP2 is a blocker of ORAI1/SOCE, and is known to activate non-excitable cells such as lymphocytes [37]. YM-58483 was reported as a potential therapeutic drug for airway inflammation in bronchial asthma and lung ischemia-reperfusion injury [38, 39]. However, YM-58483 is not a specific inhibitor of ORAI1; therefore, we established an ORAI1 gene knockdown model. Pharmacologic and genetic inhibition of ORAI1 showed the same result, strongly suggesting that ORAI1 plays a role in DOX-induced cardiac toxicity. Considering the limitation of this study, we did not establish ORAI1 gene knockdown mice. We showed that DOX increased apoptosis and YM-58483 attenuated it in vivo. Although there are no SOCE inhibitors currently used clinically, administering SOCE inhibitors might be optimal for preventing DOX-induced heart failure.

In conclusion, ORAI1/SOCE regulates p53 expression and plays an important role in DOX-induced cardiotoxicity in HCFs. ORAI1 may be a new target for preventing DOX-induced heart failure.

## Supporting information

S1 Fig Cell cycle assays for evaluating the effects of SOCE inhibition. There were four groups: the CTRL group, DOX group, YM group, and YM+DOX group. DOX decreased the proportion of cells in the G1 and S phases. In contrast, DOX increased the proportion of cells in the G2 phase. This suggests that DOX induced cell cycle arrest in the G2/M phase. YM-58483 attenuated the changes induced by DOX.
(TIF)

S2 Fig Relationship between DOX, p53, and ORAI1 in CFs. DOX increased the expression of p53 and induced apoptosis, cell cycle arrest, and ROS production. In addition, DOX increased the expression of ORAI1, not STIM1. Furthermore, the inhibition of ORAI1 negated the DOX-induced expression of p53, suggesting that the DOX-ORAI1-p53 pathway induces cardiotoxicity.
(TIF)

S1 Table Protocol for western blotting. (DOCX)

S1 Raw images Raw images of western blotting in Figs 1–3. (PDF)

S1 File Original data (data shown in Fig 1). (PDF)

S2 File Original data (data shown in Fig 2). (PDF)

S3 File Original data (data shown in Fig 3). (PDF)

S4 File Original data (data shown in Fig 4). (PDF)

S5 File Original data (data shown in Fig 5). (PDF)

S6 File Original data (data shown in Fig 6). (PDF)
