# Supplementary figures and images for: Store-operated calcium entry via ORAI1 regulates doxorubicin-induced apoptosis and prevents cardiotoxicity in cardiac fibroblasts
Source: PLoS One. 2022 Dec 6;17(12):e0278613. doi: 10.1371/journal.pone.0278613 (PMC9725120; doi:10.1371/journal.pone.0278613)

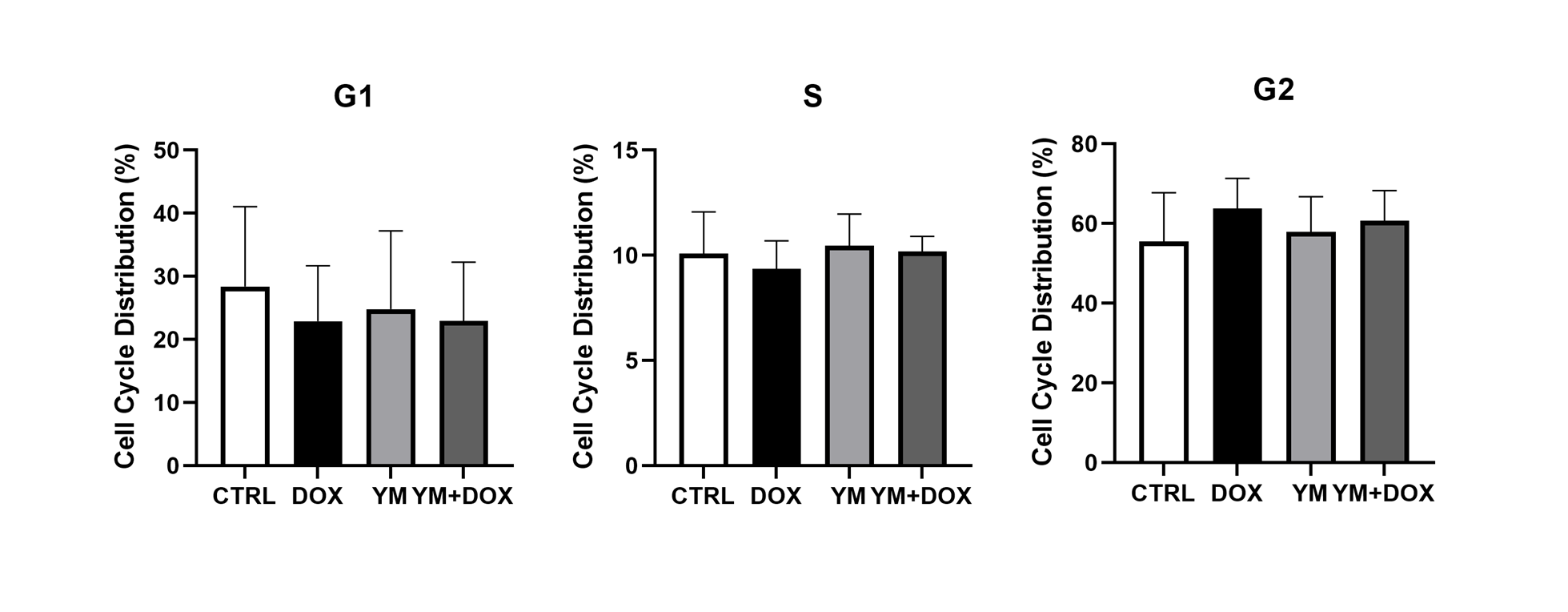

Supplement: S1 Fig — There were four groups: the CTRL group, DOX group, YM group, and YM+DOX group. DOX decreased the proportion of cells in the G1 and S phases. In contrast, DOX increased the proportion of cells in the G2 phase. This suggests that DOX induced cell cycle arrest in the G2/M phase. YM-58483 attenuated the changes induced by DOX. (TIF) [file pone.0278613.s001.tif]

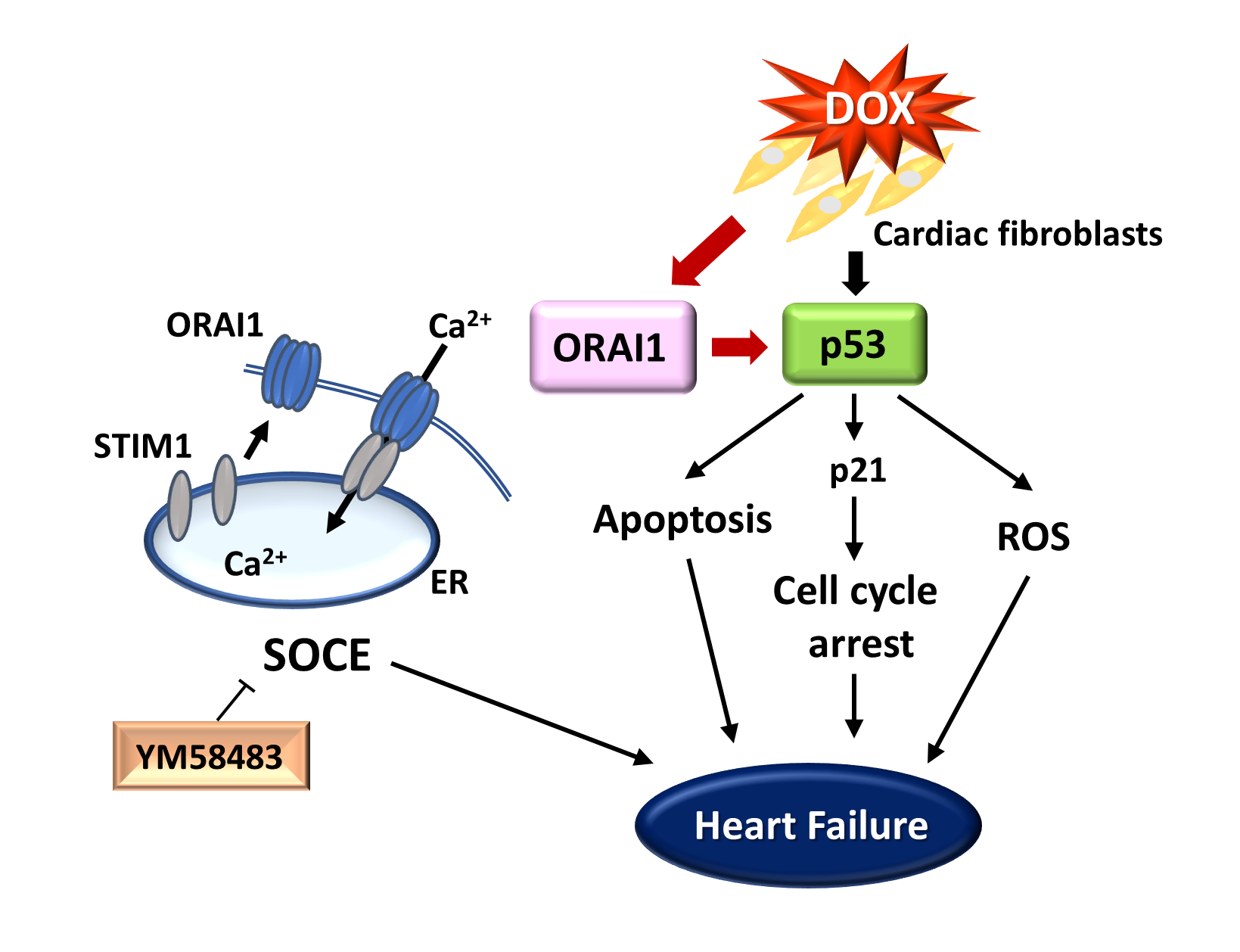

Supplement: S2 Fig — DOX increased the expression of p53 and induced apoptosis, cell cycle arrest, and ROS production. In addition, DOX increased the expression of ORAI1, not STIM1. Furthermore, the inhibition of ORAI1 negated the DOX-induced expression of p53, suggesting that the DOX-ORAI1-p53 pathway induces cardiotoxicity. (TIF) [file pone.0278613.s002.tif]

Figure1

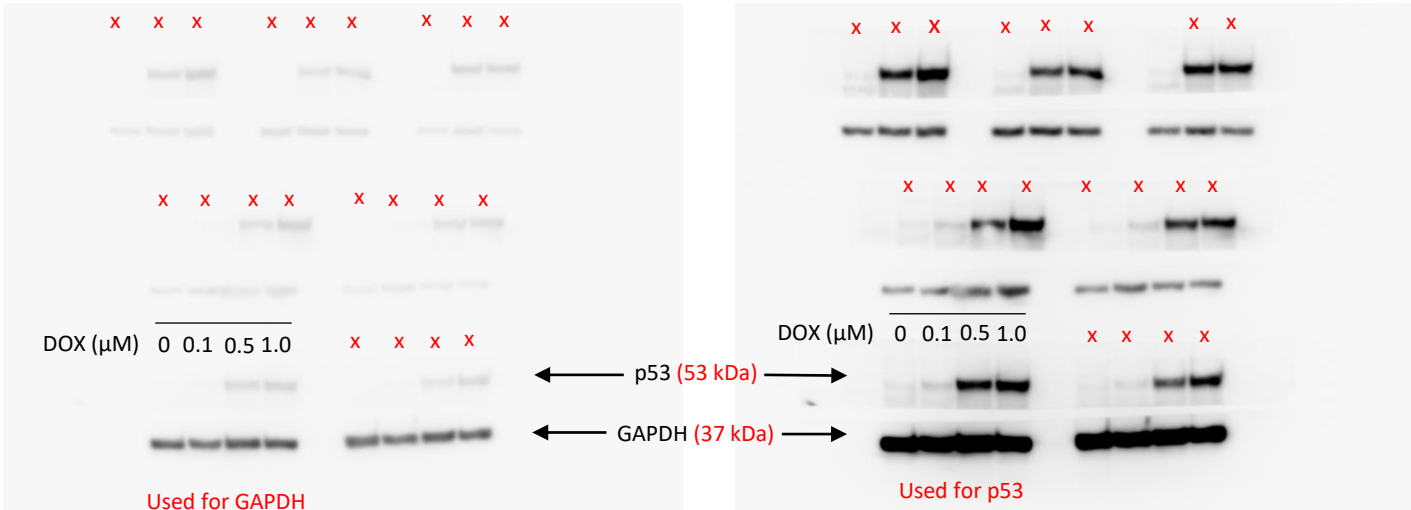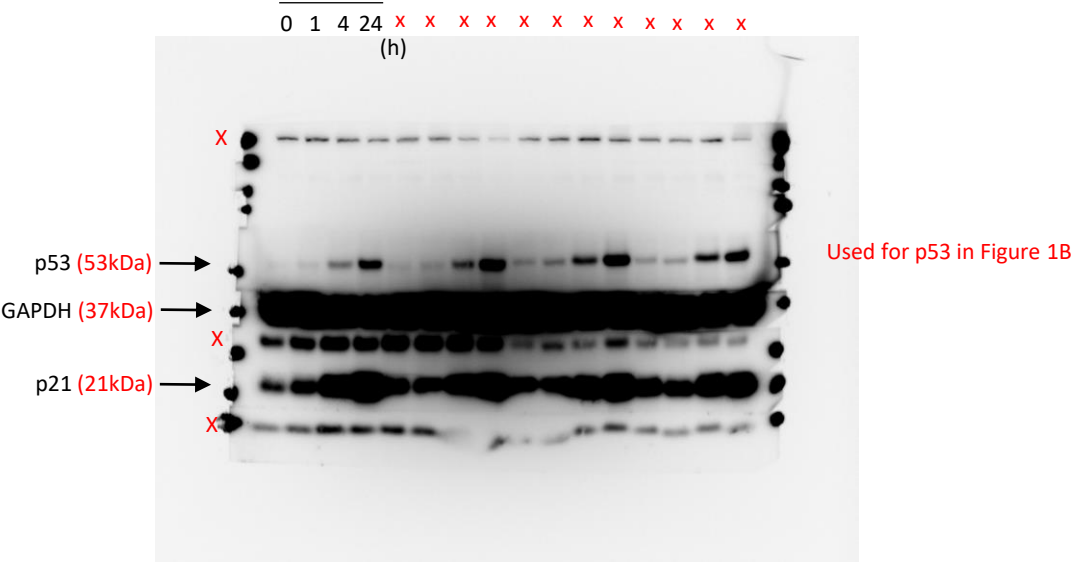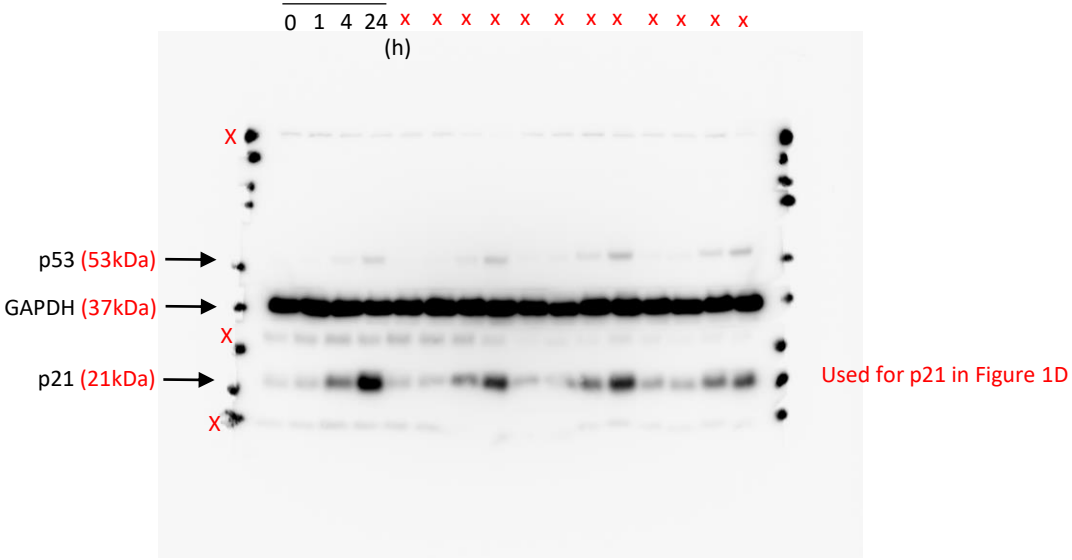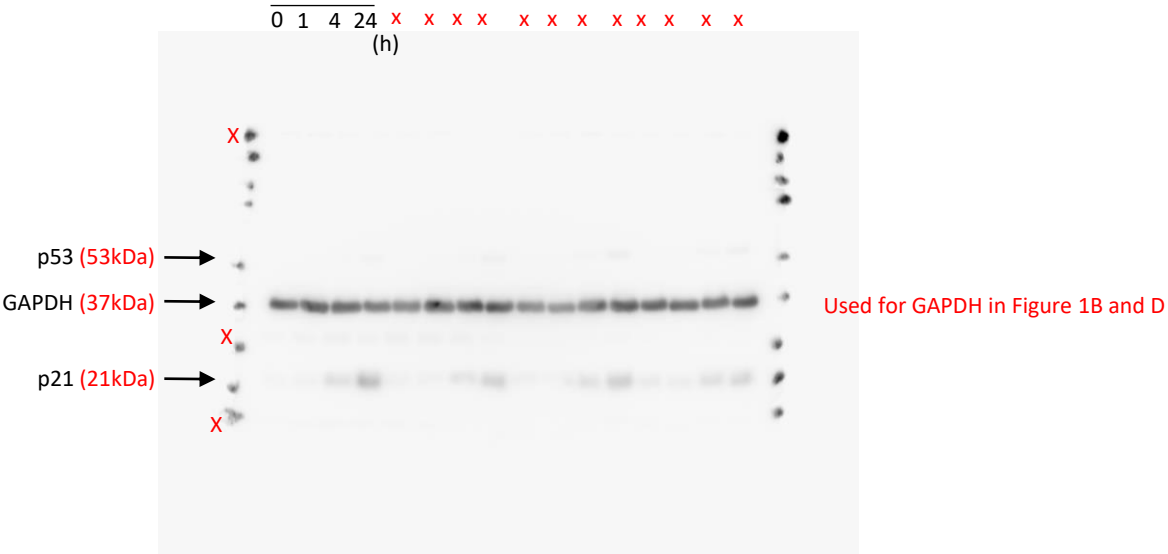

Figure 2

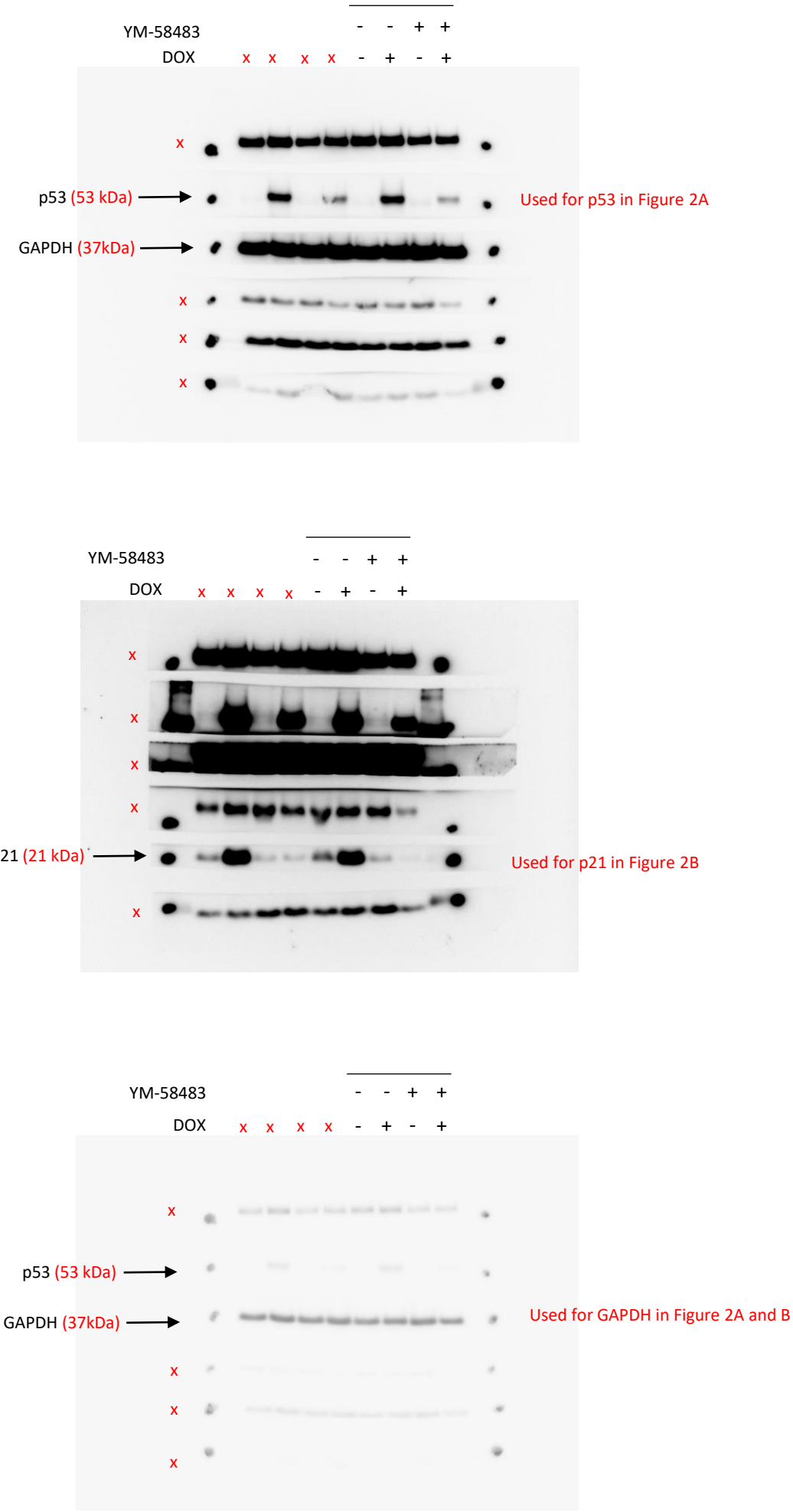

Figure 3

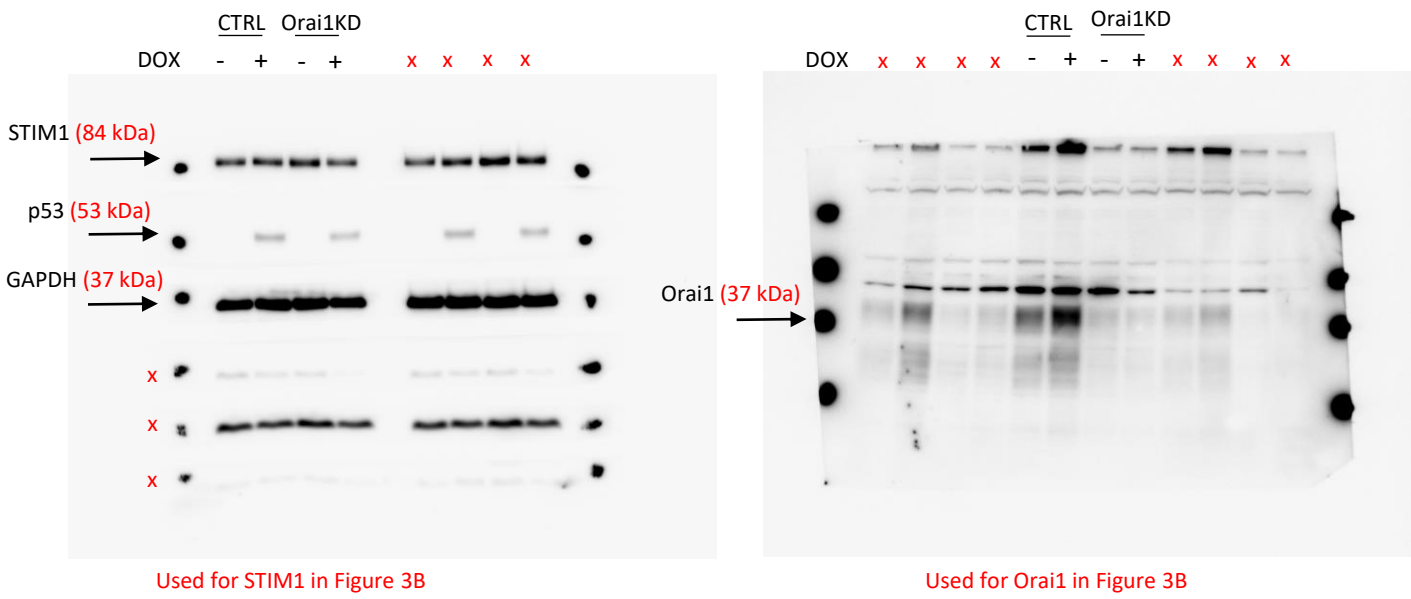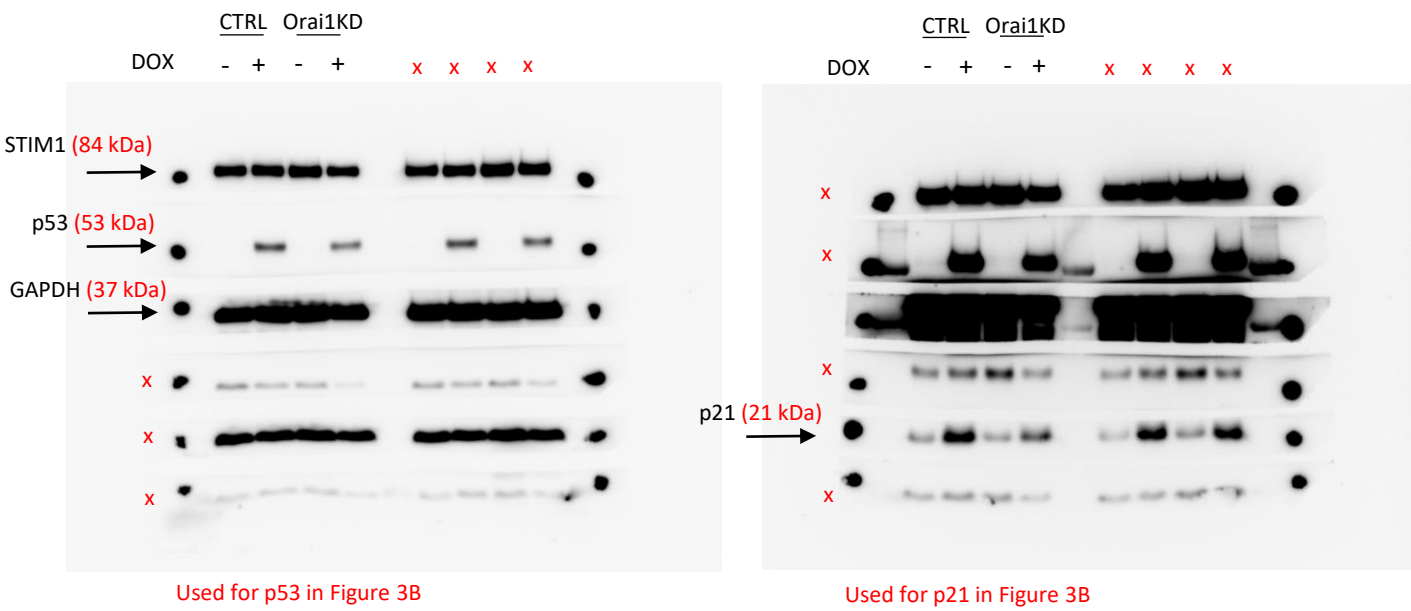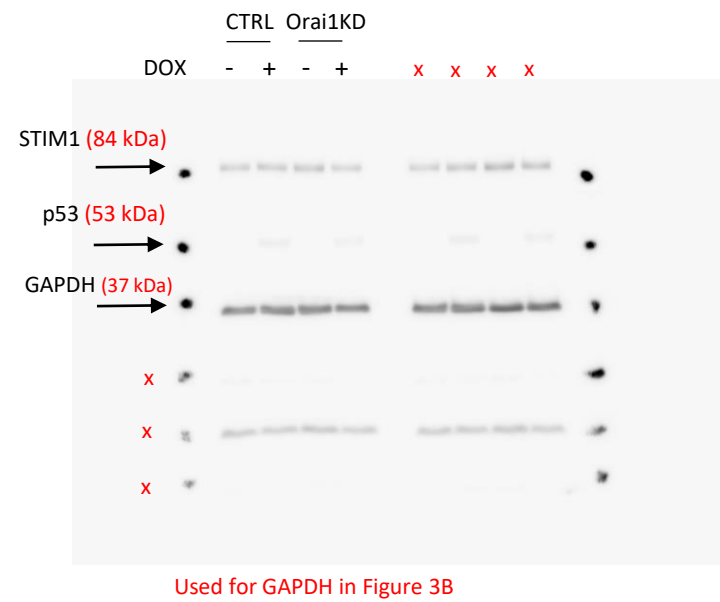

Supplement: S1 Raw images — (PDF) [file pone.0278613.s004.pdf]
