# Supplementary material for: Store-operated calcium entry via ORAI1 regulates doxorubicin-induced apoptosis and prevents cardiotoxicity in cardiac fibroblasts
Source: PLoS One. 2022 Dec 6;17(12):e0278613. doi: 10.1371/journal.pone.0278613 (PMC9725120; doi:10.1371/journal.pone.0278613)
Supplement: S1 Table — (DOCX) [file pone.0278613.s003.docx]

Protocol for Western blotting

1. Prepare separating and stacking gels. 6% separating gels are used for STIM1, p53, p21 and GAPDH. 10% separating gel is for Orai1.

|  | Separating Gel | | Stacking Gel |
| --- | --- | --- | --- |
|  | 6% | 10% |  |
| 40% Acrylamide/BIS (38:2) | 2.25 ml | 3.75 ml | 375 μl |
| 1.5 M Tris-HCl (pH 8.8) | 3.75 ml | 3.75 ml | － |
| 1 M Tris-HCl (pH 6.8) | － | － | 625 μl |
| 10% SDS | 150 μl | 150 μl | 50 μl |
| DDW | 8.73 ml | 7.23 ml | 3.89 ml |
| 10% APS | 120 ml | 120 ml | 50 μl |
| TEMED | 6 μl | 6 μl | 10 μl |

1. Apply 10 μg equivalent samples to each well.
2. For loading of 6% gel, use 150 V and 55 min running with AllView PAGE Buffer. For 10% gel, use 120 V and 90 min running with 1× loading buffer.

Composition of 1× loading buffer:

| Tris | 45.45 g |
| --- | --- |
| Glycine | 216.3 g |
| SDS | 15 g |
| DDW |  |
| fill up to | 15 L |

1. Protein bands were transferred to a Millipore Immobilon-P membrane. For transfer, use 200 mA (for 2 gels) and 120 min running.

Composition of transfer buffer:

| Tris | 30.3 g |
| --- | --- |
| Glycine | 144 g |
| DDW |  |
| fill up to | 2 L |

+ Methanol 2 L + DDW 6 L = Total 10 L

1. For STIM1, p53, p21 and GAPDH, 0.3% bovine serum albumin in 0.1% TBS-T is used for 45 min for blocking. For Orai1, 5% skim milk in 0.1% TBS-T is used for 30 min for blocking.

Composition of 5x TBS

| Tris | 391.5 g |
| --- | --- |
| NaCl | 302.5 g |
| DW |  |
| pH adjustment pH 7.6 (HCl) |  |
| fill up to | 10 L |

Composition of 0.1% TBS-T (1× TBS-T)

| 5× TBS | 1.5 L |
| --- | --- |
| Tween 20 | 7.5 ml |
| DDW |  |
| fill up to | 7.5 L |

1. Washing with 0.1% TBS-T for 5min for 3 times.
2. Primary antibodies for 15 hours in 4℃: anti-STIM1 (1:1000), anti-ORAI1 (1:1000), anti-p53 (1:1000), anti-p21 (1:1000), and anti- GAPDH (1:4000) in 0.1% TBS-T.
3. Washing with 0.1% TBS-T for 5min for 3 times.
4. Secondary antibodies for 1 hour. 1:4000 for use against STIM1, p53, p21 and GAPDH antibodies, and 1:2000 for use against ORAI1 antibody in 0.1% TBS-T.
5. Washing with 0.1% TBS-T for 5min for 3 times.
6. Chemiluminescence detection using ECL reagent.
