# Supplementary material for: Store-operated calcium entry via ORAI1 regulates doxorubicin-induced apoptosis and prevents cardiotoxicity in cardiac fibroblasts
Source: PLoS One. 2022 Dec 6;17(12):e0278613. doi: 10.1371/journal.pone.0278613 (PMC9725120; doi:10.1371/journal.pone.0278613)
Supplement: S1 File — (PDF) [file pone.0278613.s005.pdf]

Fig. 1A

CTRL	DOX 0.1 µM	DOX 0.5 µM	DOX 1.0 µM
1.25333728	1.93402501	3.520393824	6.158190814
0.74666273	1.054588414	3.520393824	6.158190814
0.97461832	2.056895703	6.42036401	8.626283642
1.02538168	1.415547391	4.122885893	7.625831058

Table Analyzed	p53/GAPDH				
Data sets analyzed	A-D				
ANOVA summary					
F	35.16				
P value	<0.0001				
P value summary	****				
Significant diff. among means (P < 0.05)?	Yes				
R squared	0.8979				
Brown-Forsythe test					
F (DFn, DFd)	1.596 (3, 12)				
P value	0.2419				
P value summary	ns				
Are SDs significantly different (P < 0.05)?	No				
Bartlett's test					
Bartlett's statistic (corrected)	8.562				
P value	0.0357				
P value summary	*				
Are SDs significantly different (P < 0.05)?	Yes				
ANOVA table	SS	DF	MS	F (DFn, DFd)	P value
Treatment (between columns)	95.46	3	31.82	F (3, 12) = 35.16	P<0.0001
Residual (within columns)	10.86	12	0.905		
Total	106.3	15			
Data summary					
Number of treatments (columns)	4				
Number of values (total)	16				

Number of families	1					
Number of comparisons per family	6					
Alpha	0.05					
Tukey's multiple comparisons test	Mean Diff.	95.00% CI of diff.	Below threshold?	Summary	Adjusted P Value	
CTRL vs. DOX 0.1 µM	-0.6153	-2.612 to 1.382	No	ns	0.7976	A-B
CTRL vs. DOX 0.5 µM	-3.396	-5.393 to -1.399	Yes	**	0.0014	A-C
CTRL vs. DOX 1.0 µM	-6.142	-8.139 to -4.145	Yes	****	<0.0001	A-D
DOX 0.1 µM vs. DOX 0.5 µM	-2.781	-4.778 to -0.7837	Yes	**	0.0066	B-C
DOX 0.1 µM vs. DOX 1.0 µM	-5.527	-7.524 to -3.530	Yes	****	<0.0001	B-D
DOX 0.5 µM vs. DOX 1.0 µM	-2.746	-4.743 to -0.7490	Yes	**	0.0072	C-D

Test details	Mean 1	Mean 2	Mean Diff.	SE of diff.	n1	n2	q	DF
CTRL vs. DOX 0.1 µM	1	1.615	-0.6153	0.6727	4	4	1.294	12
CTRL vs. DOX 0.5 µM	1	4.396	-3.396	0.6727	4	4	7.14	12
CTRL vs. DOX 1.0 µM	1	7.142	-6.142	0.6727	4	4	12.91	12
DOX 0.1 µM vs. DOX 0.5 µM	1.615	4.396	-2.781	0.6727	4	4	5.846	12
DOX 0.1 µM vs. DOX 1.0 µM	1.615	7.142	-5.527	0.6727	4	4	11.62	12
DOX 0.5 µM vs. DOX 1.0 µM	4.396	7.142	-2.746	0.6727	4	4	5.773	12

Fig. 1B

CTRL	1 h	4 h	24 h
0.579649	0.60395	0.871794	1.626929
1.079344	1.068397	1.540939	2.130345
1.443407	1.481085	1.445751	1.655494
0.8976	0.719416	1.134532	1.476389

Table Analyzed	p53				
Data sets analyzed	A-D				
ANOVA summary					
F	4.228				
P value	0.0295				
P value summary	*				
Significant diff. among means (P < 0.05)?	Yes				
R squared	0.5139				
Brown-Forsythe test					
F (DFn, DFd)	0.3584 (3, 12)				
P value	0.7841				
P value summary	ns				
Are SDs significantly different (P < 0.05)?	No				
Bartlett's test					
Bartlett's statistic (corrected)	0.3632				
P value	0.9477				
P value summary	ns				
Are SDs significantly different (P < 0.05)?	No				
ANOVA table	SS	DF	MS	F (DFn, DFd)	P value
Treatment (between columns)	1.456	3	0.4854	F (3, 12) = 4.228	P=0.0295
Residual (within columns)	1.378	12	0.1148		
Total	2.834	15			
Data summary					
Number of treatments (columns)	4				
Number of values (total)	16				

Number of families	1								
Number of comparisons per family	6								
Alpha	0.05								
Tukey's multiple comparisons test	Mean Diff.	95.00% CI of diff.	Below threshold?	Summary	Adjusted P Value				
CTRL vs. 1 h	0.03179	-0.6795 to 0.7431	No	ns	0.9991	A-B			
CTRL vs. 4 h	-0.2483	-0.9596 to 0.4630	No	ns	0.7323	A-C			
CTRL vs. 24 h	-0.7223	-1.434 to -0.01099	Yes	*	0.0462	A-D			
1 h vs. 4 h	-0.28	-0.9913 to 0.4313	No	ns	0.6565	B-C			
1 h vs. 24 h	-0.7541	-1.465 to -0.04278	Yes	*	0.0367	B-D			
4 h vs. 24 h	-0.474	-1.185 to 0.2373	No	ns	0.2488	C-D			
Test details	Mean 1	Mean 2	Mean Diff.	SE of diff.	n1	n2	q	DF	
CTRL vs. 1 h	1	0.9682	0.03179	0.2396	4	4	0.1876	12	
CTRL vs. 4 h	1	1.248	-0.2483	0.2396	4	4	1.465	12	
CTRL vs. 24 h	1	1.722	-0.7223	0.2396	4	4	4.264	12	
1 h vs. 4 h	0.9682	1.248	-0.28	0.2396	4	4	1.653	12	
1 h vs. 24 h	0.9682	1.722	-0.7541	0.2396	4	4	4.451	12	
4 h vs. 24 h	1.248	1.722	-0.474	0.2396	4	4	2.798	12	

Fig. 1C

CTRL	DOX
0.66	3.15
0.382	2.55
0.819	5.48
1.82	4.46

Table Analyzed	Q3
Column B	DOX
vs.	vs.
Column A	CTRL
Unpaired t test	
P value	0.0063
P value summary	**
Significantly different (P < 0.05)?	Yes
One- or two-tailed P value?	Two-tailed
t, df	t=4.106, df=6
How big is the difference?	
Mean of column A	0.9195
Mean of column B	3.91
Difference between means (B - A) ± SEM	2.991 ± 0.7284
95% confidence interval	1.208 to 4.773
R squared (eta squared)	0.7375
F test to compare variances	
F, DFn, Dfd	4.433, 3, 3
P value	0.2528
P value summary	ns
Significantly different (P < 0.05)?	No
Data analyzed	
Sample size, column A	4
Sample size, column B	4

Fig. 1D

CTRL	1 h	4 h	24 h
0.567291	0.736277	1.966846	4.890958
1.043162	0.794312	1.673745	3.171931
1.14015	1.221814	1.826695	3.024736
1.249397	0.983311	2.023655	2.503143

Table Analyzed	p21				
Data sets analyzed	A-D				
ANOVA summary					
F	17.03				
P value	0.0001				
P value summary	***				
Significant diff. among means (P < 0.05)?	Yes				
R squared	0.8098				
Brown-Forsythe test					
F (DFn, DFd)	1.246 (3, 12)				
P value	0.3365				
P value summary	ns				
Are SDs significantly different (P < 0.05)?	No				
Bartlett's test					
Bartlett's statistic (corrected)	11.55				
P value	0.0091				
P value summary	**				
Are SDs significantly different (P < 0.05)?	Yes				
ANOVA table	SS	DF	MS	F (DFn, DFd)	P value
Treatment (between columns)	15.79	3	5.264	F (3, 12) = 17.03	P=0.0001
Residual (within columns)	3.708	12	0.309		
Total	19.5	15			
Data summary					
Number of treatments (columns)	4				
Number of values (total)	16				

Number of families	1					
Number of comparisons per family	6					
Alpha	0.05					
Tukey's multiple comparisons test	Mean Diff.	95.00% CI of diff.	Below threshold?	Summary	Adjusted P Value	
CTRL vs. 1 h	0.06607	-1.101 to 1.233	No	ns	0.9982	A-B
CTRL vs. 4 h	-0.8727	-2.040 to 0.2943	No	ns	0.1729	A-C
CTRL vs. 24 h	-2.398	-3.565 to -1.231	Yes	***	0.0003	A-D
1 h vs. 4 h	-0.9388	-2.106 to 0.2282	No	ns	0.1325	B-C
1 h vs. 24 h	-2.464	-3.631 to -1.297	Yes	***	0.0002	B-D
4 h vs. 24 h	-1.525	-2.692 to -0.3579	Yes	*	0.0102	C-D

Test details	Mean 1	Mean 2	Mean Diff.	SE of diff.	n1	n2	q	DF
CTRL vs. 1 h	1	0.9339	0.06607	0.3931	4	4	0.2377	12
CTRL vs. 4 h	1	1.873	-0.8727	0.3931	4	4	3.14	12
CTRL vs. 24 h	1	3.398	-2.398	0.3931	4	4	8.626	12
1 h vs. 4 h	0.9339	1.873	-0.9388	0.3931	4	4	3.378	12
1 h vs. 24 h	0.9339	3.398	-2.464	0.3931	4	4	8.864	12
4 h vs. 24 h	1.873	3.398	-1.525	0.3931	4	4	5.486	12

Fig. 1E

G1

CTRL	DOX
86.2	80.8
83.4	79.2
83.1	83.9
84.7	81.3
82.9	76.7
85.6	78.7

Table Analyzed	G1
Column B	DOX
vs.	vs.
Column A	CTRL
Unpaired t test	
P value	0.0046
P value summary	**
Significantly different (P < 0.05)?	Yes
One- or two-tailed P value?	Two-tailed
t, df	t=3.635, df=10
How big is the difference?	
Mean of column A	84.32
Mean of column B	80.1
Difference between means (B - A) ± SEM	-4.217 ± 1.160
95% confidence interval	-6.801 to -1.632
R squared (eta squared)	0.5692
F test to compare variances	
F, DFn, Dfd	3.175, 5, 5
P value	0.2305
P value summary	ns
Significantly different (P < 0.05)?	No
Data analyzed	
Sample size, column A	6
Sample size, column B	6

Fig. 1E

S

CTRL	DOX
2.58	2.78
2.29	6.81
2.31	2.18
4.18	1.98
1.53	2.47
1.47	3.51

Table Analyzed	S
Column B	DOX
vs.	vs.
Column A	CTRL
Unpaired t test	
P value	0.3116
P value summary	ns
Significantly different (P < 0.05)?	No
One- or two-tailed P value?	Two-tailed
t, df	t=1.066, df=10
How big is the difference?	
Mean of column A	2.393
Mean of column B	3.288
Difference between means (B - A) ± SEM	0.8950 ± 0.8399
95% confidence interval	-0.9764 to 2.766
R squared (eta squared)	0.102
F test to compare variances	
F, DFn, Dfd	3.370, 5, 5
P value	0.2085
P value summary	ns
Significantly different (P < 0.05)?	No
Data analyzed	
Sample size, column A	6
Sample size, column B	6

Fig. 1E

G2

CTRL	DOX
3.88	8.48
4.13	6.96
4.23	8.45
3.78	8.58
4.01	8.06
4.75	8.37

Table Analyzed	G2
Column B	DOX
vs.	vs.
Column A	CTRL
Unpaired t test	
P value	<0.0001
P value summary	****
Significantly different (P < 0.05)?	Yes
One- or two-tailed P value?	Two-tailed
t, df	t=14.07, df=10
How big is the difference?	
Mean of column A	4.13
Mean of column B	8.15
Difference between means (B - A) ± SEM	4.020 ± 0.2858
95% confidence interval	3.383 to 4.657
R squared (eta squared)	0.9519
F test to compare variances	
F, DFn, Dfd	3.126, 5, 5
P value	0.2366
P value summary	ns
Significantly different (P < 0.05)?	No
Data analyzed	
Sample size, column A	6
Sample size, column B	6

Fig. 1F

0	0.2	0.5	1.0	5.0
-0.2609	1.850975	3.355214	6.496897	1.626868
0.931125	1.57932	2.76995	1.372695	2.605979
1.446639	1.623897	1.512892	1.311336	2.93165
1.912857	1.521108	2.188357	1.499607	3.814265
1.318154	0.975177	1.144218	1.604493	5.42951
0.652128	1.763395	1.91041	3.226029	12.06669

Table Analyzed	Data 2				
Data sets analyzed	A-E				
ANOVA summary					
F	3.101				
P value	0.0334				
P value summary	*				
Significant diff. among means (P < 0.05)?	Yes				
R squared	0.3317				
Brown-Forsythe test					
F (DFn, DFd)	1.498 (4, 25)				
P value	0.2329				
P value summary	ns				
Are SDs significantly different (P < 0.05)?	No				
Bartlett's test					
Bartlett's statistic (corrected)	28.52				
P value	<0.0001				
P value summary	****				
Are SDs significantly different (P < 0.05)?	Yes				
ANOVA table	SS	DF	MS	F (DFn, DFd)	P value
Treatment (between columns)	49.68	4	12.42	F (4, 25) = 3.101	P=0.0334
Residual (within columns)	100.1	25	4.004		
Total	149.8	29			
Data summary					
Number of treatments (columns)	5				
Number of values (total)	30				

Number of families	1							
Number of comparisons per family	4							
Alpha	0.05							
Dunnett's multiple comparisons test	Mean Diff.	95.00% CI of diff.	Below threshold?	Summary	Adjusted P Value	A-?		
0 vs. 0.2	-0.5523	-3.564 to 2.460	No	ns	0.9688	B	0.2	
0 vs. 0.5	-1.147	-4.159 to 1.865	No	ns	0.7191	C	0.5	
0 vs. 1.0	-1.585	-4.597 to 1.427	No	ns	0.4647	D	1	
0 vs. 5.0	-3.746	-6.758 to -0.7338	Yes	*	0.0118	E	5	
Test details	Mean 1	Mean 2	Mean Diff.	SE of diff.	n1	n2	q	DF
0 vs. 0.2	1	1.552	-0.5523	1.155	6	6	0.4781	25
0 vs. 0.5	1	2.147	-1.147	1.155	6	6	0.9926	25
0 vs. 1.0	1	2.585	-1.585	1.155	6	6	1.372	25
0 vs. 5.0	1	4.746	-3.746	1.155	6	6	3.242	25
