# Supplementary material for: Store-operated calcium entry via ORAI1 regulates doxorubicin-induced apoptosis and prevents cardiotoxicity in cardiac fibroblasts
Source: PLoS One. 2022 Dec 6;17(12):e0278613. doi: 10.1371/journal.pone.0278613 (PMC9725120; doi:10.1371/journal.pone.0278613)
Supplement: S2 File — (PDF) [file pone.0278613.s006.pdf]

Fig. 2A

CTRL	DOX	YM	YM+DOX
1	11.92255	1.521215	3.189678
1	16.33618	2.165172	2.624909
0.696435	17.84737	1.303341	7.86922
1.303565	21.69771	1.16133	6.367638
0.991484	13.73275	1.063996	4.121824
1.008516	13.31499	1.056484	3.414574

Table Analyzed	p53				
Data sets analyzed	A-D				
ANOVA summary					
F		66.16			
P value	<0.0001				
P value summary	****				
Significant diff. among means (P < 0.05)?	Yes				
R squared		0.9085			
Brown-Forsythe test					
F (DFn, DFd)	5.786 (3, 20)				
P value		0.0051			
P value summary	**				
Are SDs significantly different (P < 0.05)?	Yes				
Bartlett's test					
Bartlett's statistic (corrected)		31.83			
P value	<0.0001				
P value summary	****				
Are SDs significantly different (P < 0.05)?	Yes				
ANOVA table	SS	DF	MS	F (DFn, DFd)	P value
Treatment (between columns)		865	3	288.3 F (3, 20) = 66.16	P<0.0001
Residual (within columns)		87.16	20	4.358	
Total		952.1	23		
Data summary					
Number of treatments (columns)		4			
Number of values (total)		24			

Number of families		1							
Number of comparisons per family		6							
Alpha		0.05							
Tukey's multiple comparisons test	Mean Diff.	95.00% CI of diff.	Below threshold?	Summary	Adjusted P Value				
CTRL vs. DOX	-14.81	-18.18 to -11.44	Yes	****	<0.0001	A-B			
CTRL vs. YM	-0.3786	-3.752 to 2.995	No	ns	0.9889	A-C			
CTRL vs. YM+DOX	-3.598	-6.971 to -0.2245	Yes	*	0.034	A-D			
DOX vs. YM	14.43	11.06 to 17.80	Yes	****	<0.0001	B-C			
DOX vs. YM+DOX	11.21	7.837 to 14.58	Yes	****	<0.0001	B-D			
YM vs. YM+DOX	-3.219	-6.593 to 0.1541	No	ns	0.0647	C-D			
Test details	Mean 1	Mean 2	Mean Diff.	SE of diff.	n1	n2	q	DF	
CTRL vs. DOX	1	15.81	-14.81	1.205	6	6	17.38	20	
CTRL vs. YM	1	1.379	-0.3786	1.205	6	6	0.4442	20	
CTRL vs. YM+DOX	1	4.598	-3.598	1.205	6	6	4.222	20	
DOX vs. YM	15.81	1.379	14.43	1.205	6	6	16.93	20	
DOX vs. YM+DOX	15.81	4.598	11.21	1.205	6	6	13.15	20	
YM vs. YM+DOX	1.379	4.598	-3.219	1.205	6	6	3.777	20	

Fig. 2B

CTRL	DOX	YM	YM+DOX
1	1.259944	0.729746	0.216279
1	1.95377	0.601106	0.164174
0.779653	2.874419	0.469123	0.349319
1.220347	3.13149	0.449707	0.199501
1.012125	3.23703	0.414118	0.183585
0.987875	2.625714	0.50154	0.320814

Table Analyzed	p21				
Data sets analyzed	A-D				
ANOVA summary					
F	39.3				
P value	<0.0001				
P value summary	****				
Significant diff. among means (P < 0.05)?	Yes				
R squared	0.855				
Brown-Forsythe test					
F (DFn, DFd)	5.065 (3, 20)				
P value	0.009				
P value summary	**				
Are SDs significantly different (P < 0.05)?	Yes				
Bartlett's test					
Bartlett's statistic (corrected)	29.81				
P value	<0.0001				
P value summary	****				
Are SDs significantly different (P < 0.05)?	Yes				
ANOVA table	SS	DF	MS	F (DFn, DFd)	P value
Treatment (between columns)	18.44	3	6.148	F (3, 20) = 39.30	P<0.0001
Residual (within columns)	3.129	20	0.1565		
Total	21.57	23			
Data summary					
Number of treatments (columns)	4				
Number of values (total)	24				

Number of families	1							
Number of comparisons per family	6							
Alpha	0.05							
Tukey's multiple comparisons test	Mean Diff.	95.00% CI of diff.	Below threshold?	Summary	Adjusted P Value			
CTRL vs. DOX	-1.514	-2.153 to -0.8745	Yes	****	<0.0001	A-B		
CTRL vs. YM	0.4724	-0.1668 to 1.112	No	ns	0.1973	A-C		
CTRL vs. YM+DOX	0.7611	0.1219 to 1.400	Yes	*	0.0161	A-D		
DOX vs. YM	1.986	1.347 to 2.625	Yes	****	<0.0001	B-C		
DOX vs. YM+DOX	2.275	1.636 to 2.914	Yes	****	<0.0001	B-D		
YM vs. YM+DOX	0.2886	-0.3506 to 0.9278	No	ns	0.5954	C-D		
Test details	Mean 1	Mean 2	Mean Diff.	SE of diff.	n1	n2	q	DF
CTRL vs. DOX	1	2.514	-1.514	0.2284	6	6	9.374	20
CTRL vs. YM	1	0.5276	0.4724	0.2284	6	6	2.926	20
CTRL vs. YM+DOX	1	0.2389	0.7611	0.2284	6	6	4.713	20
DOX vs. YM	2.514	0.5276	1.986	0.2284	6	6	12.3	20
DOX vs. YM+DOX	2.514	0.2389	2.275	0.2284	6	6	14.09	20
YM vs. YM+DOX	0.5276	0.2389	0.2886	0.2284	6	6	1.787	20
