# Supplementary material for: Store-operated calcium entry via ORAI1 regulates doxorubicin-induced apoptosis and prevents cardiotoxicity in cardiac fibroblasts
Source: PLoS One. 2022 Dec 6;17(12):e0278613. doi: 10.1371/journal.pone.0278613 (PMC9725120; doi:10.1371/journal.pone.0278613)
Supplement: S3 File — (PDF) [file pone.0278613.s007.pdf]

Fig. 3A

CTRL siRNA		Orai1 siRNA	
CTRL	DOX	CTRL	DOX
1.10076	2.955246	0.160713	0.337132
0.960754	2.191626	0.174661	0.321657
0.883373	3.645128	0.204033	0.37972
0.855903	4.137785	0.182464	0.34303
1.048022	2.51535	0.129987	0.354937
1.151188	3.341559	0.143167	0.631723

Table Analyzed	mRNA Orai1/18S				
Two-way ANOVA	Ordinary				
Alpha	0.05				
Source of Variation	% of total variation	P value	P value summary	Significant?	
Interaction	15.22	<0.0001	****	Yes	
Row Factor	53.62	<0.0001	****	Yes	
Column Factor	23.42	<0.0001	****	Yes	
ANOVA table	SS	DF	MS	F (DFn, DFd)	P value
Interaction	5.428	1	5.428	F (1, 20) = 39.35	P<0.0001
Row Factor	19.12	1	19.12	F (1, 20) = 138.6	P<0.0001
Column Factor	8.354	1	8.354	F (1, 20) = 60.56	P<0.0001
Residual	2.759	20	0.1379		
Difference between column means					
Mean of CTRL	0.5829				
Mean of DOX	1.763				
Difference between means	-1.18				
SE of difference	0.1516				
95% CI of difference	-1.496 to -0.8637				
Difference between row means					
Mean of CTRL siRNA	2.066				
Mean of ORAI1 siRNA	0.2803				
Difference between means	1.785				
SE of difference	0.1516				
95% CI of difference	1.469 to 2.102				
Interaction CI					
Mean diff, A1 - B1	-2.131				
Mean diff, A2 - B2	-0.2289				
(A1 -B1) - (A2 - B2)	-1.902				
95% CI of difference	-2.535 to -1.270				
(B1 - A1) - (B2 - A2)	1.902				
95% CI of difference	1.270 to 2.535				
Data summary					
Number of columns (Column Factor)	2				
Number of rows (Row Factor)	2				
Number of values	24				

Fig. 3A

Compare cell means regardless of rows and columns

Number of families	1
Number of comparisons per family	6
Alpha	0.05

Tukey's multiple comparisons test	Mean Diff.	95.00% CI of diff.	Below threshold?	Summary	Adjusted P Value
CTRL siRNA:CTRL vs. CTRL siRNA:DOX	-2.131	-2.731 to -1.531	Yes	****	<0.0001
CTRL siRNA:CTRL vs. ORAI1 siRNA:CTRL	0.8342	0.2340 to 1.434	Yes	**	0.0046
CTRL siRNA:CTRL vs. ORAI1 siRNA:DOX	0.6053	0.005128 to 1.205	Yes	*	0.0476
CTRL siRNA:DOX vs. ORAI1 siRNA:CTRL	2.965	2.365 to 3.565	Yes	****	<0.0001
CTRL siRNA:DOX vs. ORAI1 siRNA:DOX	2.736	2.136 to 3.337	Yes	****	<0.0001
ORAI1 siRNA:CTRL vs. ORAI1 siRNA:DOX	-0.2289	-0.8290 to 0.3713	No	ns	0.7127

Test details	Mean 1	Mean 2	Mean Diff.	SE of diff.	N1	N2	q	DF
CTRL siRNA:CTRL vs. CTRL siRNA:DOX	1	3.131	-2.131	0.2144	6	6	14.06	20
CTRL siRNA:CTRL vs. ORAI1 siRNA:CTRL	1	0.1658	0.8342	0.2144	6	6	5.502	20
CTRL siRNA:CTRL vs. ORAI1 siRNA:DOX	1	0.3947	0.6053	0.2144	6	6	3.992	20
CTRL siRNA:DOX vs. ORAI1 siRNA:CTRL	3.131	0.1658	2.965	0.2144	6	6	19.56	20
CTRL siRNA:DOX vs. ORAI1 siRNA:DOX	3.131	0.3947	2.736	0.2144	6	6	18.05	20
ORAI1 siRNA:CTRL vs. ORAI1 siRNA:DOX	0.1658	0.3947	-0.2289	0.2144	6	6	1.509	20

Fig. 3C

CTRL siRNA		Orai1 siRNA	
CTRL	DOX	CTRL	DOX
1	1.3230576	1.351336	1.016973
0.943691917	1.2779106	1.398505691	0.895155022
1.056308083	1.46251739	1.396624846	1.366257365
1.103855825	1.00608766	1.134178024	0.913949865
0.89614417	1.00709479	1.19901283	1.08679425
1	1.28331655	1.240866899	1.277795394
1	1.156978	1.0622159	0.9048686

Table Analyzed	STIM1 n7				
Two-way ANOVA	Ordinary				
Alpha	0.05				
Source of Variation	% of total variation	P value	P value summary	Significant?	
Interaction	34.71	0.0013	**	Yes	
Row Factor	2.281	0.3599	ns	No	
Column Factor	0.1656	0.8036	ns	No	
ANOVA table	SS	DF	MS	F (DFn, DFd)	P value
Interaction	0.2876	1	0.2876	F (1, 24) = 13.25	P=0.0013
Row Factor	0.01891	1	0.01891	F (1, 24) = 0.8712	P=0.3599
Column Factor	0.001372	1	0.001372	F (1, 24) = 0.06323	P=0.8036
Residual	0.5208	24	0.0217		
Difference between column means					
Mean of CTRL	1.127				
Mean of DOX	1.141				
Difference between means	-0.014				
SE of difference	0.05568				
95% CI of difference	-0.1289 to 0.1009				
Difference between row means					
Mean of CTRL siRNA	1.108				
Mean of ORAI1 siRNA	1.16				
Difference between means	-0.05197				
SE of difference	0.05568				
95% CI of difference	-0.1669 to 0.06295				
Interaction CI					
Mean diff, A1 - B1	-0.2167				
Mean diff, A2 - B2	0.1887				
(A1 -B1) - (A2 - B2)	-0.4054				
95% CI of difference	-0.6353 to -0.1756				
(B1 - A1) - (B2 - A2)	0.4054				
95% CI of difference	0.1756 to 0.6353				
Data summary					
Number of columns (Column Factor)	2				
Number of rows (Row Factor)	2				
Number of values	28				

Fig. 3C

Compare cell means regardless of rows and columns

Number of families	1
Number of comparisons per family	6
Alpha	0.05

Tukey's multiple comparisons test	Mean Diff.	95.00% CI of diff.	Below threshold?	Summary	Adjusted P Value
CTRL siRNA:CTRL vs. CTRL siRNA:DOX	-0.2167	-0.4339 to 0.0005130	No	ns	0.0507
CTRL siRNA:CTRL vs. ORAI1 siRNA:CTRL	-0.2547	-0.4719 to -0.03746	Yes	*	0.0173
CTRL siRNA:CTRL vs. ORAI1 siRNA:DOX	-0.06597	-0.2832 to 0.1513	No	ns	0.836
CTRL siRNA:DOX vs. ORAI1 siRNA:CTRL	-0.03797	-0.2552 to 0.1793	No	ns	0.9623
CTRL siRNA:DOX vs. ORAI1 siRNA:DOX	0.1507	-0.06648 to 0.3680	No	ns	0.2488
ORAI1 siRNA:CTRL vs. ORAI1 siRNA:DOX	0.1887	-0.02852 to 0.4059	No	ns	0.1048

Test details	Mean 1	Mean 2	Mean Diff.	SE of diff.	N1	N2	q	DF
CTRL siRNA:CTRL vs. CTRL siRNA:DOX	1	1.217	-0.2167	0.07874	7	7	3.892	24
CTRL siRNA:CTRL vs. ORAI1 siRNA:CTRL	1	1.255	-0.2547	0.07874	7	7	4.574	24
CTRL siRNA:CTRL vs. ORAI1 siRNA:DOX	1	1.066	-0.06597	0.07874	7	7	1.185	24
CTRL siRNA:DOX vs. ORAI1 siRNA:CTRL	1.217	1.255	-0.03797	0.07874	7	7	0.6819	24
CTRL siRNA:DOX vs. ORAI1 siRNA:DOX	1.217	1.066	0.1507	0.07874	7	7	2.707	24
ORAI1 siRNA:CTRL vs. ORAI1 siRNA:DOX	1.255	1.066	0.1887	0.07874	7	7	3.389	24

Fig. 3D

CTRL siRNA		Orai1 siRNA	
CTRL	DOX	CTRL	DOX
1	2.417075	0.611647	0.710438
0.82326	2.238215	0.632902	0.616765
1.17674	2.160134	0.211146	0.483016
1	1.209992	0.35083	0.636837
1	1.902216	0.664481	0.440499
1	3.082908	0.596214	0.299175
1	1.74024	0.495396	0.220121

Table Analyzed	Orai1 n7				
Two-way ANOVA	Ordinary				
Alpha	0.05				
Source of Variation	% of total variation	P value	P value summary	Significant?	
Interaction	15.32	0.0001	***	Yes	
Row Factor	53.53	<0.0001	****	Yes	
Column Factor	14.13	0.0002	***	Yes	
ANOVA table	SS	DF	MS	F (DFn, DFd)	P value
Interaction	2.233	1	2.233	F (1, 24) = 21.59	P=0.0001
Row Factor	7.803	1	7.803	F (1, 24) = 75.46	P<0.0001
Column Factor	2.06	1	2.06	F (1, 24) = 19.92	P=0.0002
Residual	2.482	24	0.1034		
Difference between column means					
Mean of CTRL	0.7545				
Mean of DOX	1.297				
Difference between means	-0.5425				
SE of difference	0.1215				
95% CI of difference	-0.7933 to -0.2917				
Difference between row means					
Mean of CTRL siRNA	1.554				
Mean of ORAI1 siRNA	0.4978				
Difference between means	1.056				
SE of difference	0.1215				
95% CI of difference	0.8050 to 1.307				
Interaction CI					
Mean diff, A1 - B1	-1.107				
Mean diff, A2 - B2	0.02225				
(A1 -B1) - (A2 - B2)	-1.13				
95% CI of difference	-1.631 to -0.6278				
(B1 - A1) - (B2 - A2)	1.13				
95% CI of difference	0.6278 to 1.631				
Data summary					
Number of columns (Column Factor)	2				
Number of rows (Row Factor)	2				
Number of values	28				

Fig. 3D

Compare cell means regardless of rows and columns

Number of families	1
Number of comparisons per family	6
Alpha	0.05

Tukey's multiple comparisons test	Mean Diff.	95.00% CI of diff.	Below threshold?	Summary	Adjusted P Value
CTRL siRNA:CTRL vs. CTRL siRNA:DOX	-1.107	-1.581 to -0.6331	Yes	****	<0.0001
CTRL siRNA:CTRL vs. ORAI1 siRNA:CTRL	0.4911	0.01690 to 0.9652	Yes	*	0.0405
CTRL siRNA:CTRL vs. ORAI1 siRNA:DOX	0.5133	0.03916 to 0.9875	Yes	*	0.0304
CTRL siRNA:DOX vs. ORAI1 siRNA:CTRL	1.598	1.124 to 2.072	Yes	****	<0.0001
CTRL siRNA:DOX vs. ORAI1 siRNA:DOX	1.621	1.146 to 2.095	Yes	****	<0.0001
ORAI1 siRNA:CTRL vs. ORAI1 siRNA:DOX	0.02225	-0.4519 to 0.4964	No	ns	0.9992

Test details	Mean 1	Mean 2	Mean Diff.	SE of diff.	N1	N2	q	DF
CTRL siRNA:CTRL vs. CTRL siRNA:DOX	1	2.107	-1.107	0.1719	7	7	9.11	24
CTRL siRNA:CTRL vs. ORAI1 siRNA:CTRL	1	0.5089	0.4911	0.1719	7	7	4.04	24
CTRL siRNA:CTRL vs. ORAI1 siRNA:DOX	1	0.4867	0.5133	0.1719	7	7	4.223	24
CTRL siRNA:DOX vs. ORAI1 siRNA:CTRL	2.107	0.5089	1.598	0.1719	7	7	13.15	24
CTRL siRNA:DOX vs. ORAI1 siRNA:DOX	2.107	0.4867	1.621	0.1719	7	7	13.33	24
ORAI1 siRNA:CTRL vs. ORAI1 siRNA:DOX	0.5089	0.4867	0.02225	0.1719	7	7	0.1831	24

Fig. 3E

CTRL siRNA		Orai1 siRNA	
CTRL	DOX	CTRL	DOX
1	15.507033	1.0710628	7.6635841
1	7.3833833	0.9965369	4.8539647
1.1319273	11.37294183	1.1715592	4.2169503
0.8680727	9.685807558	0.8208243	0.6896666
0.9460034	17.21381362	0.9960812	13.755517
0.9460034	17.2138136	0.9960812	13.755517
1	10.2195808	1.5498629	7.0792648
1	9.5695643	0.9373045	7.3451155

Table Analyzed	p53 n8					
Two-way ANOVA	Ordinary					
Alpha	0.05					
Source of Variation	% of total variation	P value	P value summary		Significant?	
Interaction	5.059	0.0257	*		Yes	
Row Factor	4.733	0.0305	*		Yes	
Column Factor	64.71	<0.0001	****		Yes	
ANOVA table	SS	DF	MS		F (DFn, DFd)	P value
Interaction	48.64	1	48.64		F (1, 28) = 5.555	P=0.0257
Row Factor	45.5	1	45.5		F (1, 28) = 5.196	P=0.0305
Column Factor	622.1	1	622.1		F (1, 28) = 71.04	P<0.0001
Residual	245.2	28	8.757			
Difference between column means						
Mean of CTRL	1.027					
Mean of DOX	9.845					
Difference between means	-8.818					
SE of difference	1.046					
95% CI of difference	-10.96 to -6.675					
Difference between row means						
Mean of CTRL siRNA	6.629					
Mean of ORAI1 siRNA	4.244					
Difference between means	2.385					
SE of difference	1.046					
95% CI of difference	0.2418 to 4.528					
Interaction CI						
Mean diff, A1 - B1	-11.28					
Mean diff, A2 - B2	-6.353					
(A1 -B1) - (A2 - B2)	-4.932					
95% CI of difference	-9.218 to -0.6455					
(B1 - A1) - (B2 - A2)	4.932					
95% CI of difference	0.6455 to 9.218					
Data summary						
Number of columns (Column Factor)	2					
Number of rows (Row Factor)	2					
Number of values	32					

Fig. 3E

Compare cell means regardless of rows and columns

Number of families	1
Number of comparisons per family	6
Alpha	0.05

Tukey's multiple comparisons test	Mean Diff.	95.00% CI of diff.	Below threshold?	Summary	Adjusted P Value
CTRL siRNA:CTRL vs. CTRL siRNA:DOX	-11.28	-15.32 to -7.244	Yes	****	<0.0001
CTRL siRNA:CTRL vs. ORAI1 siRNA:CTRL	-0.08091	-4.121 to 3.959	No	ns	>0.9999
CTRL siRNA:CTRL vs. ORAI1 siRNA:DOX	-6.433	-10.47 to -2.394	Yes	***	0.0009
CTRL siRNA:DOX vs. ORAI1 siRNA:CTRL	11.2	7.164 to 15.24	Yes	****	<0.0001
CTRL siRNA:DOX vs. ORAI1 siRNA:DOX	4.851	0.8110 to 8.891	Yes	*	0.014
ORAI1 siRNA:CTRL vs. ORAI1 siRNA:DOX	-6.353	-10.39 to -2.313	Yes	**	0.001

Test details	Mean 1	Mean 2	Mean Diff.	SE of diff.	N1	N2	q	DF
CTRL siRNA:CTRL vs. CTRL siRNA:DOX	0.9865	12.27	-11.28	1.48	8	8	10.79	28
CTRL siRNA:CTRL vs. ORAI1 siRNA:CTRL	0.9865	1.067	-0.08091	1.48	8	8	0.07734	28
CTRL siRNA:CTRL vs. ORAI1 siRNA:DOX	0.9865	7.42	-6.433	1.48	8	8	6.149	28
CTRL siRNA:DOX vs. ORAI1 siRNA:CTRL	12.27	1.067	11.2	1.48	8	8	10.71	28
CTRL siRNA:DOX vs. ORAI1 siRNA:DOX	12.27	7.42	4.851	1.48	8	8	4.636	28
ORAI1 siRNA:CTRL vs. ORAI1 siRNA:DOX	1.067	7.42	-6.353	1.48	8	8	6.072	28

Fig. 3F

CTRL siRNA		Orai1 siRNA	
CTRL	DOX	CTRL	DOX
1	2.331832	0.690253	1.490733
1	8.060504	1.864066	5.808035
1.155607	2.212781	1.521396	0.310673
0.844393	1.656095	1.322123	1.270344
1.173868	3.902274	1.000958	2.628122
0.826132	3.391135	1.139625	3.338675
1	5.565525	1.216766	5.410359
1	2.754518	0.97867	2.558148

Table Analyzed	p21 n8				
Two-way ANOVA	Ordinary				
Alpha	0.05				
Source of Variation	% of total variation	P value	P value summary	Significant?	
Interaction	2.398	0.2944	ns	No	
Row Factor	0.8797	0.5228	ns	No	
Column Factor	37.9	0.0002	***	Yes	
ANOVA table	SS	DF	MS	F (DFn, DFd)	P value
Interaction	2.416	1	2.416	F (1, 28) = 1.142	P=0.2944
Row Factor	0.8864	1	0.8864	F (1, 28) = 0.4188	P=0.5228
Column Factor	38.18	1	38.18	F (1, 28) = 18.04	P=0.0002
Residual	59.26	28	2.117		
Difference between column means					
Mean of CTRL	1.108				
Mean of DOX	3.293				
Difference between means	-2.185				
SE of difference	0.5144				
95% CI of difference	-3.238 to -1.131				
Difference between row means					
Mean of CTRL siRNA	2.367				
Mean of ORAI1 siRNA	2.034				
Difference between means	0.3329				
SE of difference	0.5144				
95% CI of difference	-0.7208 to 1.386				
Interaction CI					
Mean diff, A1 - B1	-2.734				
Mean diff, A2 - B2	-1.635				
(A1 -B1) - (A2 - B2)	-1.099				
95% CI of difference	-3.206 to 1.008				
(B1 - A1) - (B2 - A2)	1.099				
95% CI of difference	-1.008 to 3.206				
Data summary					
Number of columns (Column Factor)	2				
Number of rows (Row Factor)	2				
Number of values	32				

Fig. 3F

Compare cell means regardless of rows and columns

Number of families	1
Number of comparisons per family	6
Alpha	0.05

Tukey's multiple comparisons test	Mean Diff.	95.00% CI of diff.	Below threshold?	Summary	Adjusted P Value
CTRL siRNA:CTRL vs. CTRL siRNA:DOX	-2.734	-4.720 to -0.7482	Yes	**	0.0042
CTRL siRNA:CTRL vs. ORAI1 siRNA:CTRL	-0.2167	-2.203 to 1.769	No	ns	0.9906
CTRL siRNA:CTRL vs. ORAI1 siRNA:DOX	-1.852	-3.838 to 0.1342	No	ns	0.0743
CTRL siRNA:DOX vs. ORAI1 siRNA:CTRL	2.518	0.5315 to 4.504	Yes	**	0.0089
CTRL siRNA:DOX vs. ORAI1 siRNA:DOX	0.8824	-1.104 to 2.869	No	ns	0.6239
ORAI1 siRNA:CTRL vs. ORAI1 siRNA:DOX	-1.635	-3.621 to 0.3509	No	ns	0.1351

Test details	Mean 1	Mean 2	Mean Diff.	SE of diff.	N1	N2	q	DF
CTRL siRNA:CTRL vs. CTRL siRNA:DOX	1	3.734	-2.734	0.7274	8	8	5.316	28
CTRL siRNA:CTRL vs. ORAI1 siRNA:CTRL	1	1.217	-0.2167	0.7274	8	8	0.4214	28
CTRL siRNA:CTRL vs. ORAI1 siRNA:DOX	1	2.852	-1.852	0.7274	8	8	3.6	28
CTRL siRNA:DOX vs. ORAI1 siRNA:CTRL	3.734	1.217	2.518	0.7274	8	8	4.895	28
CTRL siRNA:DOX vs. ORAI1 siRNA:DOX	3.734	2.852	0.8824	0.7274	8	8	1.716	28
ORAI1 siRNA:CTRL vs. ORAI1 siRNA:DOX	1.217	2.852	-1.635	0.7274	8	8	3.179	28
