# Supplementary material for: Store-operated calcium entry via ORAI1 regulates doxorubicin-induced apoptosis and prevents cardiotoxicity in cardiac fibroblasts
Source: PLoS One. 2022 Dec 6;17(12):e0278613. doi: 10.1371/journal.pone.0278613 (PMC9725120; doi:10.1371/journal.pone.0278613)
Supplement: S4 File — (PDF) [file pone.0278613.s008.pdf]

Fig. 4A

CTRL	DOX	YM	YM+DOX
1.82	18.03	1.66	5.48
2	7.57	0.3	1.68
2.87	13.57	2.91	9.33
1.17	15.9	2.03	4.35
2.16	13.11	2.2	9.81
2.78	10.14	1.34	7.66

Table Analyzed	apoptosis DOX_YM				
Data sets analyzed	A-D				
ANOVA summary					
F	26.16				
P value	<0.0001				
P value summary	****				
Significant diff. among means (P < 0.05)?	Yes				
R squared	0.7969				
Brown-Forsythe test					
F (DFn, DFd)	4.677 (3, 20)				
P value	0.0124				
P value summary	*				
Are SDs significantly different (P < 0.05)?	Yes				
Bartlett's test					
Bartlett's statistic (corrected)	16.61				
P value	0.0009				
P value summary	***				
Are SDs significantly different (P < 0.05)?	Yes				
ANOVA table	SS	DF	MS	F (DFn, DFd)	P value
Treatment (between columns)	497.3	3	165.8	F (3, 20) = 26.16	P<0.0001
Residual (within columns)	126.7	20	6.337		
Total	624	23			
Data summary					
Number of treatments (columns)	4				
Number of values (total)	24				

Number of families	1							
Number of comparisons per family	6							
Alpha	0.05							
Tukey's multiple comparisons test	Mean Diff.	95.00% CI of diff.	Below threshold?	Summary	Adjusted P Value			
CTRL vs. DOX	-10.92	-14.99 to -6.852	Yes	****	<0.0001	A-B		
CTRL vs. YM	0.3933	-3.674 to 4.461	No	ns	0.9928	A-C		
CTRL vs. YM+DOX	-4.252	-8.319 to -0.1839	Yes	*	0.0385	A-D		
DOX vs. YM	11.31	7.246 to 15.38	Yes	****	<0.0001	B-C		
DOX vs. YM+DOX	6.668	2.601 to 10.74	Yes	***	0.0009	B-D		
YM vs. YM+DOX	-4.645	-8.713 to -0.5772	Yes	*	0.0217	C-D		
Test details	Mean 1	Mean 2	Mean Diff.	SE of diff.	n1	n2	q	DF
CTRL vs. DOX	2.133	13.05	-10.92	1.453	6	6	10.63	20
CTRL vs. YM	2.133	1.74	0.3933	1.453	6	6	0.3827	20
CTRL vs. YM+DOX	2.133	6.385	-4.252	1.453	6	6	4.137	20
DOX vs. YM	13.05	1.74	11.31	1.453	6	6	11.01	20
DOX vs. YM+DOX	13.05	6.385	6.668	1.453	6	6	6.489	20
YM vs. YM+DOX	1.74	6.385	-4.645	1.453	6	6	4.52	20

Fig. 4B

CTRL siRNA		Orai1 siRNA	
CTRL	DOX	CTRL	DOX
1.57	12.85	3.16	9.18
2.14	15.18	2.14	12.5
0.66	12.05	0.25	10.32
0.66	12.05	0.25	10.32
3.02	13.95	5.51	10.65
1.99	15.26	1.06	8.88

Table Analyzed	CTRL_Orai1KD				
Two-way ANOVA	Ordinary				
Alpha	0.05				
Source of Variation	% of total variation	P value	P value summary	Significant?	
Interaction	2.901	0.0069	**	Yes	
Row Factor	1.795	0.0282	*	Yes	
Column Factor	88.89	<0.0001	****	Yes	
ANOVA table	SS	DF	MS	F (DFn, DFd)	P value
Interaction	19.84	1	19.84	F (1, 20) = 9.050	P=0.0069
Row Factor	12.27	1	12.27	F (1, 20) = 5.599	P=0.0282
Column Factor	607.8	1	607.8	F (1, 20) = 277.3	P<0.0001
Residual	43.84	20	2.192		
Difference between column means					
Mean of CTRL	1.868				
Mean of DOX	11.93				
Difference between means	-10.06				
SE of difference	0.6044				
95% CI of difference	-11.33 to -8.804				
Difference between row means					
Mean of CTRL siRNA	7.615				
Mean of ORAI1 siRNA	6.185				
Difference between means	1.43				
SE of difference	0.6044				
95% CI of difference	0.1693 to 2.691				
Interaction CI					
Mean diff, A1 - B1	-11.88				
Mean diff, A2 - B2	-8.246				
(A1 -B1) - (A2 - B2)	-3.637				
95% CI of difference	-6.158 to -1.115				
(B1 - A1) - (B2 - A2)	3.637				
95% CI of difference	1.115 to 6.158				
Data summary					
Number of columns (Column Factor)	2				
Number of rows (Row Factor)	2				
Number of values	24				

Fig. 4B

Compare cell means regardless of rows and columns

Number of families	1
Number of comparisons per family	6
Alpha	0.05

Šídák's multiple comparisons test	Mean Diff.	95.00% CI of diff.	Below threshold?	Summary	Adjusted P Value
CTRL siRNA:CTRL vs. CTRL siRNA:DOX	-11.88	-14.38 to -9.389	Yes	****	<0.0001
CTRL siRNA:CTRL vs. ORAI1 siRNA:CTRL	-0.3882	-2.882 to 2.106	No	ns	0.9983
CTRL siRNA:CTRL vs. ORAI1 siRNA:DOX	-8.635	-11.13 to -6.141	Yes	****	<0.0001
CTRL siRNA:DOX vs. ORAI1 siRNA:CTRL	11.49	9.001 to 13.99	Yes	****	<0.0001
CTRL siRNA:DOX vs. ORAI1 siRNA:DOX	3.248	0.7545 to 5.742	Yes	**	0.0067
ORAI1 siRNA:CTRL vs. ORAI1 siRNA:DOX	-8.246	-10.74 to -5.753	Yes	****	<0.0001

Test details	Mean 1	Mean 2	Mean Diff.	SE of diff.	N1	N2	t	DF
CTRL siRNA:CTRL vs. CTRL siRNA:DOX	1.674	13.56	-11.88	0.8547	6	6	13.9	20
CTRL siRNA:CTRL vs. ORAI1 siRNA:CTRL	1.674	2.062	-0.3882	0.8547	6	6	0.4541	20
CTRL siRNA:CTRL vs. ORAI1 siRNA:DOX	1.674	10.31	-8.635	0.8547	6	6	10.1	20
CTRL siRNA:DOX vs. ORAI1 siRNA:CTRL	13.56	2.062	11.49	0.8547	6	6	13.45	20
CTRL siRNA:DOX vs. ORAI1 siRNA:DOX	13.56	10.31	3.248	0.8547	6	6	3.8	20
ORAI1 siRNA:CTRL vs. ORAI1 siRNA:DOX	2.062	10.31	-8.246	0.8547	6	6	9.648	20
