# Supplementary material for: Store-operated calcium entry via ORAI1 regulates doxorubicin-induced apoptosis and prevents cardiotoxicity in cardiac fibroblasts
Source: PLoS One. 2022 Dec 6;17(12):e0278613. doi: 10.1371/journal.pone.0278613 (PMC9725120; doi:10.1371/journal.pone.0278613)
Supplement: S5 File — (PDF) [file pone.0278613.s009.pdf]

Fig. 5A

CTRL	DOX	YM	YM+DOX
1.070921	1.315401	0.212106	0.344052
0.910961	1.550562	0.461065	0.472175
0.844241	1.442867	0.214435	1.089497
1.259552	1.566511	0.122091	0.299851
0.903972	1.569975	0.193828	0.233788
1.010353	1.009756	0.483644	0.318845

Table Analyzed	Data 2				
Data sets analyzed	A-D				
ANOVA summary					
F	32.6				
P value	<0.0001				
P value summary	****				
Significant diff. among means (P < 0.05)?	Yes				
R squared	0.8302				
Brown-Forsythe test					
F (DFn, DFd)	0.1921 (3, 20)				
P value	0.9005				
P value summary	ns				
Are SDs significantly different (P < 0.05)?	No				
Bartlett's test					
Bartlett's statistic (corrected)	3.672				
P value	0.2991				
P value summary	ns				
Are SDs significantly different (P < 0.05)?	No				
ANOVA table	SS	DF	MS	F (DFn, DFd)	P value
Treatment (between columns)	4.773	3	1.591	F (3, 20) = 32.60	P<0.0001
Residual (within columns)	0.9761	20	0.0488		
Total	5.749	23			
Data summary					
Number of treatments (columns)	4				
Number of values (total)	24				

Number of families	1
Number of comparisons per family	6
Alpha	0.05

Tukey's multiple comparisons test	Mean Diff.	95.00% CI of diff.	Below threshold?	Summary	Adjusted P Value	
CTRL vs. DOX	-0.4092	-0.7662 to -0.05218	Yes	*	0.0211	A-B
CTRL vs. YM	0.7188	0.3618 to 1.076	Yes	****	<0.0001	A-C
CTRL vs. YM+DOX	0.5403	0.1833 to 0.8973	Yes	**	0.0021	A-D
DOX vs. YM	1.128	0.7710 to 1.485	Yes	****	<0.0001	B-C
DOX vs. YM+DOX	0.9495	0.5925 to 1.306	Yes	****	<0.0001	B-D
YM vs. YM+DOX	-0.1785	-0.5355 to 0.1785	No	ns	0.5141	C-D

Test details	Mean 1	Mean 2	Mean Diff.	SE of diff.	n1	n2	q	DF
CTRL vs. DOX	1	1.409	-0.4092	0.1275	6	6	4.537	20
CTRL vs. YM	1	0.2812	0.7188	0.1275	6	6	7.97	20
CTRL vs. YM+DOX	1	0.4597	0.5403	0.1275	6	6	5.991	20
DOX vs. YM	1.409	0.2812	1.128	0.1275	6	6	12.51	20
DOX vs. YM+DOX	1.409	0.4597	0.9495	0.1275	6	6	10.53	20
YM vs. YM+DOX	0.2812	0.4597	-0.1785	0.1275	6	6	1.979	20

Fig. 5B

CTRL siRNA		Orai1 siRNA	
CTRL	DOX	CTRL	DOX
1.013335	1.376778	1.156244	1.221668
0.837889	1.198527	0.975609	1.095728
1.019996	1.251329	0.973576	1.122655
1.032198	1.210027	0.992088	1.149442
1.009268	1.384772	1.264653	1.152107
1.087314	1.184293	1.10246	1.21164

Table Analyzed	Data 4				
Two-way ANOVA	Ordinary				
Alpha	0.05				
Source of Variation	% of total variation	P value	P value summary	Significant?	
Interaction	13.16	0.0188	*	Yes	
Row Factor	0.372	0.6718	ns	No	
Column Factor	46.24	0.0001	***	Yes	
ANOVA table	SS	DF	MS	F (DFn, DFd)	P value
Interaction	0.052	1	0.052	F (1, 20) = 6.539	P=0.0188
Row Factor	0.00147	1	0.00147	F (1, 20) = 0.1849	P=0.6718
Column Factor	0.1828	1	0.1828	F (1, 20) = 22.98	P=0.0001
Residual	0.159	20	0.007952		
Difference between column means					
Mean of CTRL	1.039				
Mean of DOX	1.213				
Difference between means	-0.1745				
SE of difference	0.0364				
95% CI of difference	-0.2505 to -0.09859				
Difference between row means					
Mean of CTRL siRNA	1.134				
Mean of ORAI1 siRNA	1.118				
Difference between means	0.01565				
SE of difference	0.0364				
95% CI of difference	-0.06028 to 0.09159				
Interaction CI					
Mean diff, A1 - B1	-0.2676				
Mean diff, A2 - B2	-0.08143				
(A1 -B1) - (A2 - B2)	-0.1862				
95% CI of difference	-0.3381 to -0.03431				
(B1 - A1) - (B2 - A2)	0.1862				
95% CI of difference	0.03431 to 0.3381				
Data summary					
Number of columns (Column Factor)	2				
Number of rows (Row Factor)	2				
Number of values	24				

Fig. 5B

Compare cell means regardless of rows and columns

Number of families	1
Number of comparisons per family	6
Alpha	0.05

Tukey's multiple comparisons test	Mean Diff.	95.00% CI of diff.	Below threshold?	Summary	Adjusted P Value
CTRL siRNA:CTRL vs. CTRL siRNA:DOX	-0.2676	-0.4117 to -0.1235	Yes	***	0.0002
CTRL siRNA:CTRL vs. ORAI1 siRNA:CTRL	-0.07744	-0.2215 to 0.06666	No	ns	0.4536
CTRL siRNA:CTRL vs. ORAI1 siRNA:DOX	-0.1589	-0.3030 to -0.01477	Yes	*	0.0274
CTRL siRNA:DOX vs. ORAI1 siRNA:CTRL	0.1902	0.04608 to 0.3343	Yes	**	0.0072
CTRL siRNA:DOX vs. ORAI1 siRNA:DOX	0.1087	-0.03535 to 0.2528	No	ns	0.1833
ORAI1 siRNA:CTRL vs. ORAI1 siRNA:DOX	-0.08144	-0.2255 to 0.06266	No	ns	0.4108

Test details	Mean 1	Mean 2	Mean Diff.	SE of diff.	N1	N2	q	DF
CTRL siRNA:CTRL vs. CTRL siRNA:DOX	1	1.268	-0.2676	0.05148	6	6	7.351	20
CTRL siRNA:CTRL vs. ORAI1 siRNA:CTRL	1	1.077	-0.07744	0.05148	6	6	2.127	20
CTRL siRNA:CTRL vs. ORAI1 siRNA:DOX	1	1.159	-0.1589	0.05148	6	6	4.364	20
CTRL siRNA:DOX vs. ORAI1 siRNA:CTRL	1.268	1.077	0.1902	0.05148	6	6	5.224	20
CTRL siRNA:DOX vs. ORAI1 siRNA:DOX	1.268	1.159	0.1087	0.05148	6	6	2.987	20
ORAI1 siRNA:CTRL vs. ORAI1 siRNA:DOX	1.077	1.159	-0.08144	0.05148	6	6	2.237	20
