# Supplementary material for: Store-operated calcium entry via ORAI1 regulates doxorubicin-induced apoptosis and prevents cardiotoxicity in cardiac fibroblasts
Source: PLoS One. 2022 Dec 6;17(12):e0278613. doi: 10.1371/journal.pone.0278613 (PMC9725120; doi:10.1371/journal.pone.0278613)
Supplement: S6 File — (PDF) [file pone.0278613.s010.pdf]

Fig. 6B

CTRL	DOX	YM	YM+DOX
0.038948	0.859071	0.037175	0.440529
0.062176	0.691742	0.01747	0.485437
0.088889	0.612423	0.038454	0.411722
0.067904	0.49473	0.056904	0.355207
0.039417	1.105811	0.082169	0.392157
0.042753	0.878294	0.094003	0.475172
0.094913	0.956572	0.056022	0.338802

Table Analyzed	20220209 TUNEL/DAPI(%)				
Data sets analyzed	A-D				
ANOVA summary					
F	71.4				
P value	<0.0001				
P value summary	****				
Significant diff. among means (P < 0.05)?	Yes				
R squared	0.8992				
Brown-Forsythe test					
F (DFn, DFd)	6.990 (3, 24)				
P value	0.0015				
P value summary	**				
Are SDs significantly different (P < 0.05)?	Yes				
Bartlett's test					
Bartlett's statistic (corrected)	33.79				
P value	<0.0001				
P value summary	****				
Are SDs significantly different (P < 0.05)?	Yes				
ANOVA table	SS	DF	MS	F (DFn, DFd)	P value
Treatment (between columns)	2.628	3	0.8758	F (3, 24) = 71.40	P<0.0001
Residual (within columns)	0.2944	24	0.01227		
Total	2.922	27			
Data summary					
Number of treatments (columns)	4				
Number of values (total)	28				

Number of families	1								
Number of comparisons per family	6								
Alpha	0.05								
Tukey's multiple comparisons test	Mean Diff.	95.00% CI of diff.	Below threshold?	Summary	Adjusted P Value				
CTRL vs. DOX	-0.7377	-0.9010 to -0.5743	Yes	****	<0.0001	A-B			
CTRL vs. YM	0.007543	-0.1558 to 0.1709	No	ns	0.9992	A-C			
CTRL vs. YM+DOX	-0.352	-0.5153 to -0.1887	Yes	****	<0.0001	A-D			
DOX vs. YM	0.7452	0.5819 to 0.9085	Yes	****	<0.0001	B-C			
DOX vs. YM+DOX	0.3857	0.2223 to 0.5490	Yes	****	<0.0001	B-D			
YM vs. YM+DOX	-0.3595	-0.5229 to -0.1962	Yes	****	<0.0001	C-D			
Test details	Mean 1	Mean 2	Mean Diff.	SE of diff.	n1	n2	q	DF	
CTRL vs. DOX	0.06214	0.7998	-0.7377	0.0592	7	7	17.62	24	
CTRL vs. YM	0.06214	0.0546	0.007543	0.0592	7	7	0.1802	24	
CTRL vs. YM+DOX	0.06214	0.4141	-0.352	0.0592	7	7	8.409	24	
DOX vs. YM	0.7998	0.0546	0.7452	0.0592	7	7	17.8	24	
DOX vs. YM+DOX	0.7998	0.4141	0.3857	0.0592	7	7	9.213	24	
YM vs. YM+DOX	0.0546	0.4141	-0.3595	0.0592	7	7	8.589	24	
